# Pharmacological and functional similarities of the human neuropeptide Y system in *C. elegans* challenges phylogenetic views on the FLP/NPR system

**DOI:** 10.1186/s12964-019-0436-1

**Published:** 2019-09-18

**Authors:** Miron Mikhailowitsch Gershkovich, Victoria Elisabeth Groß, Anette Kaiser, Simone Prömel

**Affiliations:** 1 Faculty of Life Sciences, Institute of Biochemistry, Leipzig University, 04103 Leipzig, Germany; 2 Medical Faculty, Rudolf Schönheimer Institute of Biochemistry, Leipzig University, 04103 Leipzig, Germany

**Keywords:** Neuropeptide Y, RFamide, Neuropeptide GPCRs, *C. elegans*, *Npr*, *Flp*, Pharmacological and functional conservation, Orthology relationships, Context-specific activation

## Abstract

**Background:**

The neuropeptide Y system affects various processes, among others food intake, and is frequently discussed in the context of targeting obesity. Studies in model organisms are indispensable to enable molecular studies in a physiological context. Although the NPY system is evolutionarily conserved in all bilaterians, in the widely used model *Caenorhabditis elegans* there is controversy on the existence of NPY orthologous molecules. While the FMRFamide-like peptide (FLP)/Neuropeptide receptor-Resemblance (NPR) system in the nematode was initially suggested to be orthologous to the mammalian NPY system, later global phylogenetic studies indicate that FLP/NPR is protostome-specific.

**Methods:**

We performed a comprehensive pharmacological study of the FLP/NPR system in transfected cells in vitro, and tested for functional substitution in *C. elegans* knockout strains. Further, we phenotypically compared different *flp* loss-of-function strains. Differences between groups were compared by ANOVA and post-hoc testing (Dunnett, Bonferroni).

**Results:**

Our pharmacological analysis of the FLP/NPR system including formerly functionally uncharacterized NPY-like peptides from *C. elegans* demonstrates that G protein-coupling and ligand requirements for receptor activation are similar to the human NPY system. In vitro and in vivo analyses show cross-reactivity of NPY with the FLP/NPR system manifesting in the ability of the human GPCRs to functionally substitute FLP/NPR signaling in vivo. The high pharmacological/functional similarities enabled us to identify *C. elegans* FLP-14 as a key molecule in avoidance behavior.

**Conclusions:**

Our data demonstrate the pharmacological and functional similarities of human NPY and *C. elegans* NPR systems. This adds a novel perspective to current phylogenetic reconstructions of the neuropeptide Y system. NPY and NPR receptors are pharmacologically so similar that the human receptors can functionally compensate for the *C. elegans* ones, suggesting orthologous relationships. This is also underlined by the presence of NPY-like peptides and parallels in peptide requirements for receptor activation. Further, the results presented here highlight the potential of this knowledge for physiological as well as molecular studies on neuropeptide GPCRs such as the NPY system in the future.

## Background

The neuropeptide Y (NPY) family is an intensively studied system due to the essential roles of its members in regulating food intake in humans [1]. Consequently, its potential as target for modulating food consumption and thus, obesity, is intensely investigated [1]. Besides this capacity, the NPY system also has multiple other functions, for instance in the control of mood and anxiety or ethanol intake [2, 3]. The hallmark features of NPY and the related peptides PYY and PP are a C-terminal arginine-phenylalanine/tyrosine sequence and an amidated carboxy-terminus (RxRF/Yamide), which are essential for activation of their four cognate G protein-coupled receptors (GPCRs) in most mammals [4]. Due to the many facets of NPY, numerous insights are based on findings in model organisms (summarized in [5]). Rodent studies established a physiological function for single receptor subtypes. However, the specific roles of the receptors, their distinct cellular effectors, desensitization profiles, and the effects of specific mutations are difficult to address in rodent model systems. In this regard, a more basic model organism that enables higher throughput analyses and ideally offers spatiotemporal control would be desirable.

Such studies are possible in principle in simple model organisms, as several neuropeptidergic signaling systems are conserved in invertebrates. Indeed, a global phylogenetic analysis identified the neuropeptide F/Y (NPF/Y) system as one of nearly 30 ancient neuropeptidergic systems that can be traced back to the common ancestor of protostomes and deuterostomes (urbilaterian) [6–8]. For example, a clear NPF ortholog is found in the protostome *Drosophila melanogaster* [7, 9–11]. Likewise, in *Caenorhabditis elegans*, the receptor NPR-1 (NeuroPeptide receptor Resemblance-1) was initially assigned as NPY receptor ortholog. *C. elegans* is a valuable model organism due to its translucence and vast genetic toolbox. Importantly for studies on GPCRs, all major signaling pathways are present (G_s_, G_q/11_, G_i/o_, arrestins). The early classification of *C. elegans* NPRs as NPY receptor homologs was based on sequence similarities and their role in feeding behavior [12], and was recently supported by a study using vertebrate ancestral chromosome reconstruction [13]. However, in global phylogenetic analyses, NPRs and their ligands appear to form a protostome-specific clade that has diverged from a common bilaterian ancestor and is paralogous to the NPF/Y family, thus challenging a potential use of the nematode as a model system in this context [7, 8].

This notion is supported by genome analyses of *C. elegans,* which show a remarkably expanded repertoire of genes encoding potential NPR receptors comprising 41 members [14]. Likewise, the possible cognate ligands, FMRFamide-like peptides (FLPs), cover 31 genes (reviewed in [14]), each encoding a set of peptides yielding in total more than 70 FLPs. These can be subdivided into FMRFamide (ΩΨRFa; with Ω being an aromatic amino acid and Ψ being a hydrophobic amino acid) and short neuropeptides F (sNPF) harboring a C-terminal ΨΨRFa consensus. In contrast, ‘classical’ NPF/NPY sequences found in other bilateria are longer and carry a C-terminal RxRF/Yamide. Within the predicted repertoire of FLPs in *C. elegans*, FLP-27, FLP-33, FLP-34-1 and FLP-34-2 share the greatest similarity with vertebrate NPY and display this C-terminal RxRF/Ya signature. However, only FLP-27 and 33 have been biochemically isolated [15, 16] and no function has yet been assigned to these peptides. These observations leave important open questions such as whether NPF/Y orthologs exist in nematodes or have been lost in this phylum, and if so, by which mechanisms their essential physiological functions are compensated.

Here, we demonstrate that the nematode NPR/FLP system is pharmacologically and functionally highly similar to the human NPY system, and the same set of receptors is activated by FLPs of FMRF, sNPF and (long)NPF-type, adding a new perspective to current phylogenetic reconstructions. This resemblance is so profound that human NPY GPCRs are even able to phenotypically rescue NPR function in vivo in *C. elegans*. The functional homology of both neuropeptide systems was subsequently utilized to identify FLP-14 as a context-specific driver for avoidance behavior in *C. elegans*.

## Methods

### Materials

All standard chemicals were purchased from Sigma Aldrich or Carl-Roth GmbH unless stated otherwise. Cell culture materials were obtained from Lonza and enzymes from ThermoFisher Scientific.

### Cell culture

In all in vitro assays the commercially available cell line HEK293 (*Homo sapiens*, female, embryonic kidney, DSMZ ACC 305) was used. Cell line identity was confirmed by short tandem repeat profiling at eight different *loci* (performed by the Leibnitz Institut DSMZ, Braunschweig), and cells were negative for mycoplasma contamination in routine testings. Cells were maintained as a monolayer at 37 °C and 5% CO_2_ under humidified atmosphere in Dulbecco’s modified Eagle’s medium (DMEM) with Ham’s F12 (1:1; v/v) supplemented with 15% (v/v) heat inactivated fetal calf serum (FCS).

### *C. elegans* strains

*C. elegans* were maintained at 22 °C using standard conditions [17]. Wild-type worms were *C. elegans* variety Bristol, N2. Strains used in this study are listed in Additional file 1: Table S1. Strains not generated in this study were obtained from the *Caenorhabditis Genetics Center* (CGC) funded by NIH Office of Research Infrastructure Programs (P40 OD010440).

### Peptide synthesis

C-terminally amidated peptides were synthesized in 15 μmol scale by solid-phase peptide synthesis following the Fmoc/*tert*-butyl strategy (reviewed in [18]) on Rink amide resin using an automated Syro II peptide synthesizer (MultiSynTech). Automated coupling reactions were performed as double couplings using 8 eq Nα-protected amino acid, activated in situ with equimolar amounts of Oxyma and diisocarbodiimide in dimethylformamide (DMF) for 30 min. Automated Fmoc deprotection was carried out with 40% (v/v) piperidine in DMF for 3 min and 20% (v/v) piperidine in DMF for 10 min. Reactive side chains of amino acids were protected by *tert*-butyl (*t*Bu for Tyr, Ser, Asp, Glu, Thr), trityl (Trt for Asn, Gln, His), 2,2,4,6,7-pentamethyldihydrobenzofuran-5-sulfonyl (Pbf for Arg) and *tert*-butyloxycarbonyl (Boc for Lys). Peptides were cleaved off the resin using trifluoroacetic acid (TFA)/H_2_O/triisopropylsilane (90/5/5, v/v/v) for 2.5 h at room temperature and precipitated in ice cold diethyl ether. All peptides were purified to > 95% purity by RP-HPLC (Shimadzu) using a Phenomenex Jupiter 10 μm Proteo 90 Å (C12) column. For most peptides, linear gradients of H_2_O + 0.1% trifluoroacetic acid (TFA) (eluent A) and acetonitrile (ACN) + 0.08% TFA (eluent B) were applied at a flow rate of 10 ml/minute. Some crude peptides were partly insoluble (FLP-3-4, FLP-15-2, FLP-18-5, FLP-21, FLP-27, FLP-33, FLP-34-1, FLP-34-2) under these conditions. FLP-27, FLP-33, and FLP-34 were solubilized by ultrasonication at 50 °C for 10 min and subsequently purified under standard solvent conditions used pre-heated HPLC systems. The other peptides (FLP-3-4, FLP-15-2, FLP-18-5, and FLP-21) displayed improved solubility at basic pH, and were thus purified accordingly using a linear gradient of 10 mM (NH_4_)_2_CO_3_ in H_2_O (eluent A, pH 8.5) and 10 mM (NH_4_)_2_CO_3_ in 80% ACN (eluent B, pH 8.5) at a flow rate of 10 ml/minute. Peptide identity was verified by matrix-assisted laser desorption/time of flight (MALDI-ToF; Ultraflex III MALDI-ToF/ToF, Bruker Daltonics) mass spectrometry, and peptide purity was evaluated with analytical RP-HPLC.

### Generation of plasmids and transgenes

#### Neuropeptide GPCR constructs for in vitro analysis

Expression vectors encoding human NPY and RFamide receptors were kind gifts from A. G. Beck-Sickinger (Y_1_R, Y_2_R, Y_4_R, Y_5_R, NPFF_1_R, NPFF_2_R, QRFPR and PrRPR). These receptor constructs are preceded by a 5′ Kozak sequence (GCCACC) and at 3′ fused to an enhanced yellow fluorescent protein (eYFP) via an ADPPVV linker (GCGGATCCACCGGTCGTG, containing *BamH*I and *Age*I restriction sites). *C. elegans* receptor sequences (*npr-3*, *npr-6*) were amplified from *C. elegans* cDNA. For cDNA generation, a mixed population of N2 hermaphrodites was harvested, washed in M9 buffer and incubated in TRIzol reagent (ThermoFisher Scientific) for total RNA isolation according to manufacturer’s protocol. Reverse transcriptase and oligo-dT primers were used to generate cDNA. cDNA sequences of *npr-1*, *npr-4b*, *npr-5b*, and *npr-11* were purchased (GenScript). The coding sequences were fused to a 5′ Kozak sequence, and 3′ DPPVV linker in analogy to the expression vectors for human receptors using the NPR(x)_Mlu_f and NPR(x)_Linker_r primers. In a second step, these sequences were fused to an eYFP coding sequence (amplified from the Y_2_R-eYFP_pV2 parent construct with the primers Linker-YFP-f and YFP-XbaI-NheI-r) using the PCR overlap extension technique [19]. The resulting DNA fragments were ligated into the pVitro2-hygro-mcs vector (InvivoGen) using a (5′) *Mlu*I and (3′) *Xba*I (*Nhe*I in case of *npr-1*) restriction sites. For primer sequences see Additional file 1: Table S2. Chimeric Gα_Δ6qi4myr_ [20] was a kind gift from E. Kostenis.

#### Neuropeptide GPCR constructs for transgenesis

All constructs contained the cDNA of a human neuropeptide receptor C-terminally fused to a GFP downstream of a 2 kb *npr-1* promoter in vector pPD95.79. The basis for these constructs was *npr-1p::npr-1::gfp* in pPD95.79 (kind gift of L. Ma). From this vector, the backbone was amplified using primers pPD95.79_f/ pPD95.79_r. In a second step, the *npr-1* promoter was amplified from *npr-1p::npr-1::gfp* using primers npr-1p_f/ npr-1p_r and ligated back into the obtained backbone via an *Xma*I restriction site yielding plasmid pSP131.

The human neuropeptide receptors Y_1_R, Y_2_R, Y_4_R, Y_5_R, NPFF_1_, NPFFR_2_, and PrRPR were amplified from the pVitro2 vectors described above with the primers listed in Additional file 1: Table S2 and ligated into pSP131 using *Age*I restriction sites introduced via the primers. This resulted in the following constructs: *npr-1p::Y*_*1*_*R::gfp* (pSP141), *npr-1p::Y*_*2*_*R::gfp* (pSP136), *npr-1p::Y*_*4*_*R::gfp* (pSP129), *npr-1p::Y*_*5*_*R::gfp* (pSP130), *npr-1p::NPFF*_*1*_*R::gfp* (pSP139), *npr-1p::NPFF*_*2*_*R::gfp* (pSP133) and *npr-1p::PrRPR::gfp* (pSP148). For primer sequences see Additional file 1: Table S2.

### Inositol phosphate (IP) accumulation assay

For measuring IP accumulation, HEK293 cells grown to 70–80% confluency in 6-well plates were transiently co-transfected using Metafectene Pro (2.5 μl/μg DNA; Biontex) with plasmids containing receptor and/or mock (empty pcDNA3.1) (for endogenous G_q_-coupling) using 4 μg total DNA in a ratio of 3:1 following the manufacturer’s instructions. For testing Gα_16_- and Gα_Δ6qi4myr_-coupling, plasmids encoding the corresponding Gα protein were co-transfected instead of mock. Gα_16_ is naturally promiscuous and stimulates the phospholipase C (PLC) pathway leading to IP production, while the exchange of the C-terminal four amino acids of Gα_q_ for the corresponding residues of Gα_i1_ in the chimeric Gα_Δ6qi4myr_ confers the ability to couple to G_i/o_ protein-preferring receptors but still stimulate PLC, thus specifically redirecting downstream cellular signaling [21]. 16 h post transfection, the cells were seeded into white 384-well plates at a density of 20,000 cells/well. Media was removed 24 h later and the cells were incubated with the indicated concentration of peptides for 1 h in HBSS + 20 mM LiCl. IP accumulation was measured with the HTRF-based IP-One Gq kit (Cisbio) on a microplate reader (Tecan Spark).

### cAMP reporter gene assay

Activation of G_s_ and G_i/o_ was assessed with a cAMP reporter gene assay. HEK293 cells grown to 70–80% confluency in 6-well plates were transiently co-transfected with vectors encoding the receptor and the cAMP reporter gene plasmid pGL4.29 [luc2P/CRE/Hygro] (Promega; 4 μg total, 1:1 ratio) using Metafectene Pro (3 μl/μg DNA; Biontex) following the manufacturer’s instructions. 16 h post transfection, the cells were seeded into white 384-well plates at a density of 20,000 cells/well. On the next day, the medium was removed and the cells were stimulated with 20 μl of peptide solution (or forskolin as positive control) in serum-free DMEM for 4 h. For measuring G_i/o_ activity, the peptide solutions additionally contained 1 μM forskolin to elevate cellular cAMP levels. After incubation, the luciferase substrate OneGlo in lysis buffer (Promega) was added and the luminescence was measured in a microplate reader Tecan Spark (Tecan). Data were analyzed with GraphPad Prism 5.03 and are shown as fold of basal or fold of forskolin in case of Gα_i/o_ activity. All data are displayed as mean ± SEM of at least three independent experiments performed in triplicate.

### Fluorescence microscopy

Expression and membrane localization of *C. elegans* receptor constructs fused to eYFP in human cells was assessed by fluorescence microscopy. HEK293 cells were grown to 70–80% confluency in 8-well μ-slides (Ibiditreat) and transiently transfected with 1 μg vector DNA per well using Lipofectamine2000 (ThermoFisher Scientific) following the manufacturer’s instructions. On the next day, medium was changed to OptiMEM (Invitrogen Life Technologies), nuclei were stained with 2.5 ng/μl Hoechst33342 (Sigma-Aldrich) and cells were examined using an Axiovert Observer Z1 microscope (with Apotome, Plan-Apochromat 63x/1.40 Oil DIC objective, filter sets 02 (365/420), 46 (500/535); Carl Zeiss). Images were acquired using identical exposure times and post-processing.

Expression of the human neuropeptide receptors in *C. elegans* was determined by confocal fluorescent microscopy. Nematodes were anesthetized with 125 mM sodium azide and placed on 5% agar pads immediately prior to imaging using a Leica TCS SP8 confocal microscope (HyD photo detector, Leica Microsystems).

### Generation of transgenic nematodes

Transgenic strains were generated by microinjection as described previously [22]. Constructs were injected in a mixture containing the construct of interest (10 ng/μl), a marker DNA (either *pmyo-2::mCherry* (100 ng/μl, kind gift from R. Schnabel) or *pmyo-3::mCherry::unc-54* (pCFJ104, 20 ng/μl, kind gift from E. Jorgensen [23])) and pBluescript II SK+ as stuffer DNA to reach a final concentration of 120 ng/μl DNA in total. DNA was injected into the syncytical gonad of *npr-1(ky13)* hermaphrodites (strain CX4118). Microinjection was performed by NemaMetrix Inc. The F1 generation was isolated and screened for positive progeny. Several transgenic lines with stably transmitting extrachromosomal arrays were established and analyzed for each transgene (Additional file 1: Table S1).

### Bordering behavior assay

Bordering behavior was determined as described by de Bono and Bargman [12]. In brief, 120 young adults were placed on NGM plates with a thick OP50 lawn (approximately 2.5 cm in diameter). Plates were kept at 22 °C for 2 h until scoring. Animals within a 2 mm range of the border of the lawn were counted as bordering (Fig. 3).

### MeSa (methyl salicylate) avoidance assay

The MeSa avoidance assay was performed as described previously [24] with some modifications (Fig. 3). Nematode growth medium (NGM) plates (9 cm) were divided into four equal quadrants. Young adults were washed three times with M9 and circa 50 to 120 animals were transferred in 40 μl M9 to the middle of the assay plate. After placing the worms on the plate, either a drop of 2 μl ethanol (EOH) as control or of 2 μl methyl salicylate (MeSa) were positioned on two opposite quadrants equidistant from each other (3.5 cm from the center) (adapted from [25]). To paralyze nearby animals, 2 μl of 0.5 M sodium azide were dripped on each quadrant. Plates were sealed with parafilm and incubated at 22 °C until all worms were paralyzed. For measuring avoidance, the animals on each side were counted and the avoidance index was calculated by subtracting the number of worms on the ethanol sides from the number of worms in the methyl salicylate sides and divided by the total worm count.

### Statistical analysis and matrix generation

For in vitro activation, data is presented as mean ± SEM of at least three independent experiments conducted in technical triplicate. For a given signal pathway, peptides were considered to activate a particular receptor (and thus be considered in the interaction matrix) if the signal for the highest peptide concentration tested was higher than the threshold, and statistically different from the buffer control as analyzed by a one-way ANOVA with Dunnett’s post-hoc test and *p* values < 0.05. We defined the threshold as 2-fold over the buffer control for cAMP (G_s_) and IP (G_q_) production, or 20% inhibition of forskolin-induced cAMP levels at this receptor, respectively. For the *C. elegans* NPRs, we considered the strongest response elicited by any of the peptides downstream of a particular receptor as the receptor’s maximal signal. This may or may not be the biological maximum that can be reached by the ‘true’ endogenous agonist. For the human receptors, the response to the established endogenous agonist reflected the maximal signal. Color-coding of the interaction matrix for the peptide ligands is based on the gradual activities at sub-micromolar (0.1 μM) or micromolar (10 μM) peptide concentrations as a coarse measure of peptide potency (i-v). The two lightest shades (i, ii) reflect a just-above-threshold and ~ 50% of maximal receptor signal in response to 10 μM peptide, respectively, but no response at 0.1 μM concentration (corresponding to an estimated EC_50_ of 100–10 μM assuming normal curve steepness and full agonism). The mid-grade shade (iii) reflects an above-threshold signal when stimulated with 0.1 μM of peptide but still submaximal response when stimulated with 10 μM of peptide (estimated EC_50_ ~ 1 μM). The second darkest shade (iv) indicates partial activation by 0.1 μM and full activation by 10 μM of peptide (estimated EC_50_ ~ 0.1 μM), while dark green (v) illustrates maximal receptor activation already in response to 0.1 μM of agonist (estimated EC_50_ < 0.1 μM).

Of note, there were a few peptide/receptor pairs that displayed equal responses at 0.1 and 10 μM ligand stimulation, but the response was significantly lower than the maximum signal that can be elicited downstream of the given receptor (by another peptide), indicative of high affinity partial agonism. These instances are indicated in Fig. 2 and the supplemental data tables with an asterisk (*). Importantly, our screening and analysis cannot inform about full or partial agonism for ligands with lower potency.

Similarly, for the G protein-coupling preferences, we considered a pathway to be activated if at least one peptide elevated the respective second messenger significantly above control levels using a two-way ANOVA followed by Bonferroni’s post-hoc test (variable 1: pathway, variable 2: stimulation). Color-coding of the matrix shows the pathway with highest signal (fold of basal) in dark blue, and secondary pathways in light blue.

In vivo data are reported as mean ± SD. Statistical analysis was performed using GraphPad Prism version 7.0. Data were analyzed using a one-way ANOVA with a Bonferroni post-hoc test or unpaired student’s t-test and *p* values < 0.05 were considered statistically significant. All details are given in the respective Figure legend.

## Results

### Selection of *C. elegans* RFamide peptides and potential cognate GPCRs

**Fig. 1:**
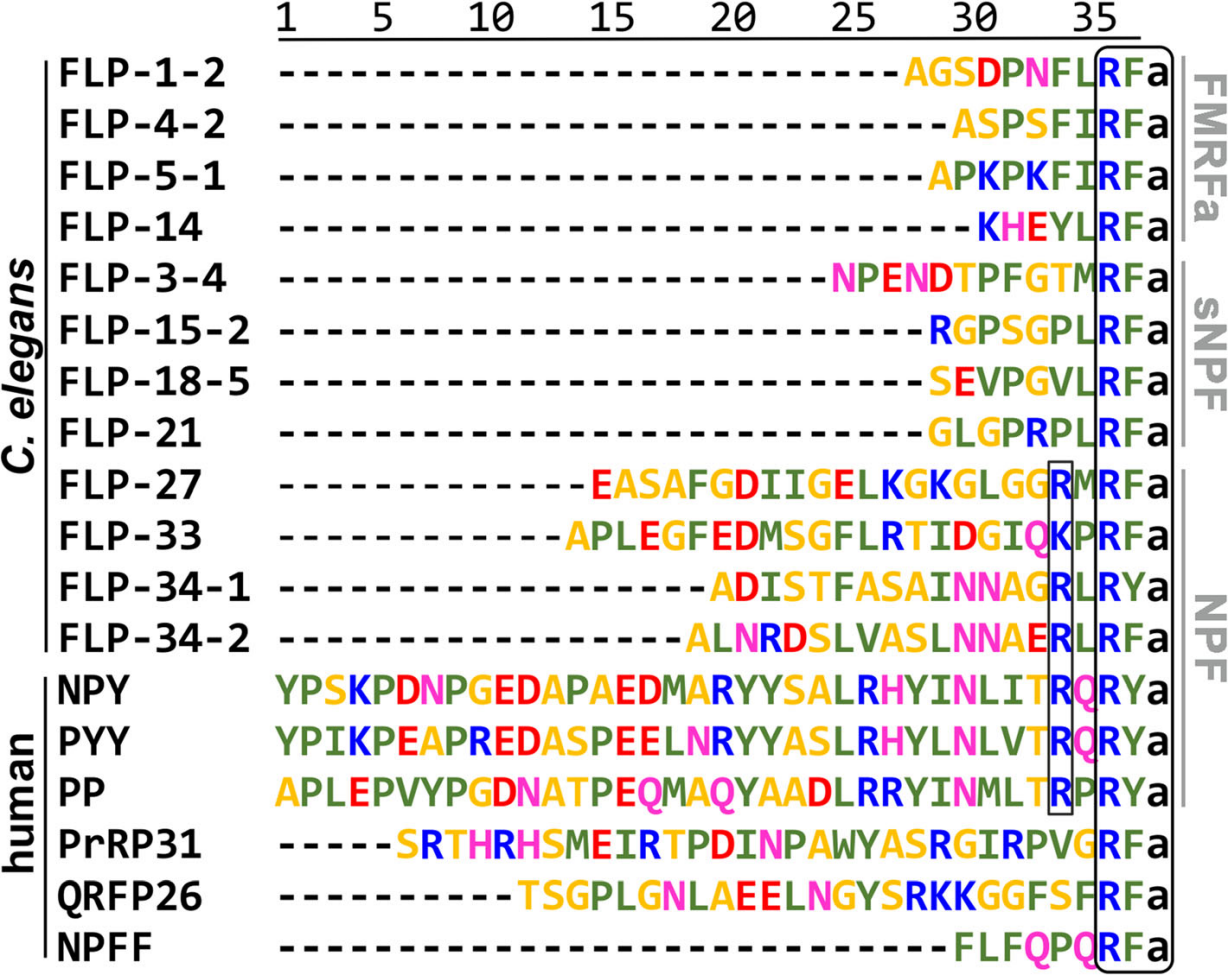
*C. elegans* and human peptide alignment. Amino acid sequences of *C. elegans* and human peptides aligned to the amidated C terminus (a-amide, conserved C-terminal RF/Ya and additional R at − 4 position highlighted in black rectangles). *C. elegans* peptide syntax consists of gene and isoform. Amino acids are colored according to their properties (Lesk scheme [26])

Global phylogenetic reconstructions indicate that the *C. elegans* NPR/FLP system is not directly orthologous to the human NPY system despite the latter being conserved in all bilaterians [6–8], and the NPR and NPY receptors display a considerable degree of homology [12]. To clarify potential similarities between these systems, we first set out to characterize the pharmacological properties of the NPR system in the roundworm and to analyze the pharmacological complementarity with the human neuropeptide NPY and related RFamide systems in vitro. For this purpose, we chose the following eight well-established FMRF- and sNPF-type FLPs, as they have been reported to activate NPR receptors [14]: FLP-1-2, FLP-3-4, FLP-4-2, FLP-5-1, FLP-14, FLP-15-2, FLP-18-5, and FLP-21 (Fig. 1). The respective isoforms were chosen to represent the average length and charge composition. Further, we included the four so far uncharacterized FLPs FLP-27, FLP-33, FLP-34-1, and FLP-34-2, which appear to be more NPY-like. All peptides were synthesized by Fmoc/*tert*-butyl solid-phase chemistry and purified to > 95% to ensure high assay confidence (Additional file 1: Table S3).

For comparing these *C. elegans* and the human neuropeptide systems in vitro*,* we selected the following six well-characterized NPRs: NPR-1, NPR-3, NPR-4b, NPR-5b, NPR-6, and NPR-11. These receptors were chosen as they were identified as most closely related to the human NPY receptors in a recent phylogenetic analysis [27]. Moreover, we considered NPR-4 to be representative also for the GPCR NPR-10 (which was not tested), NPR-5 for NPR-13, and NPR-11 for NPR-12, since these receptors show a close phylogenetic relationship and likely arose from recent gene duplication as suggested by the same study [27]. For NPR-4 and NPR-5, which have multiple isoforms originating from alternative splicing, we chose the longest variants (NPR-4b, NPR-5b).

### Human and *C. elegans* neuropeptide receptors are pharmacologically similar

**Fig. 2:**
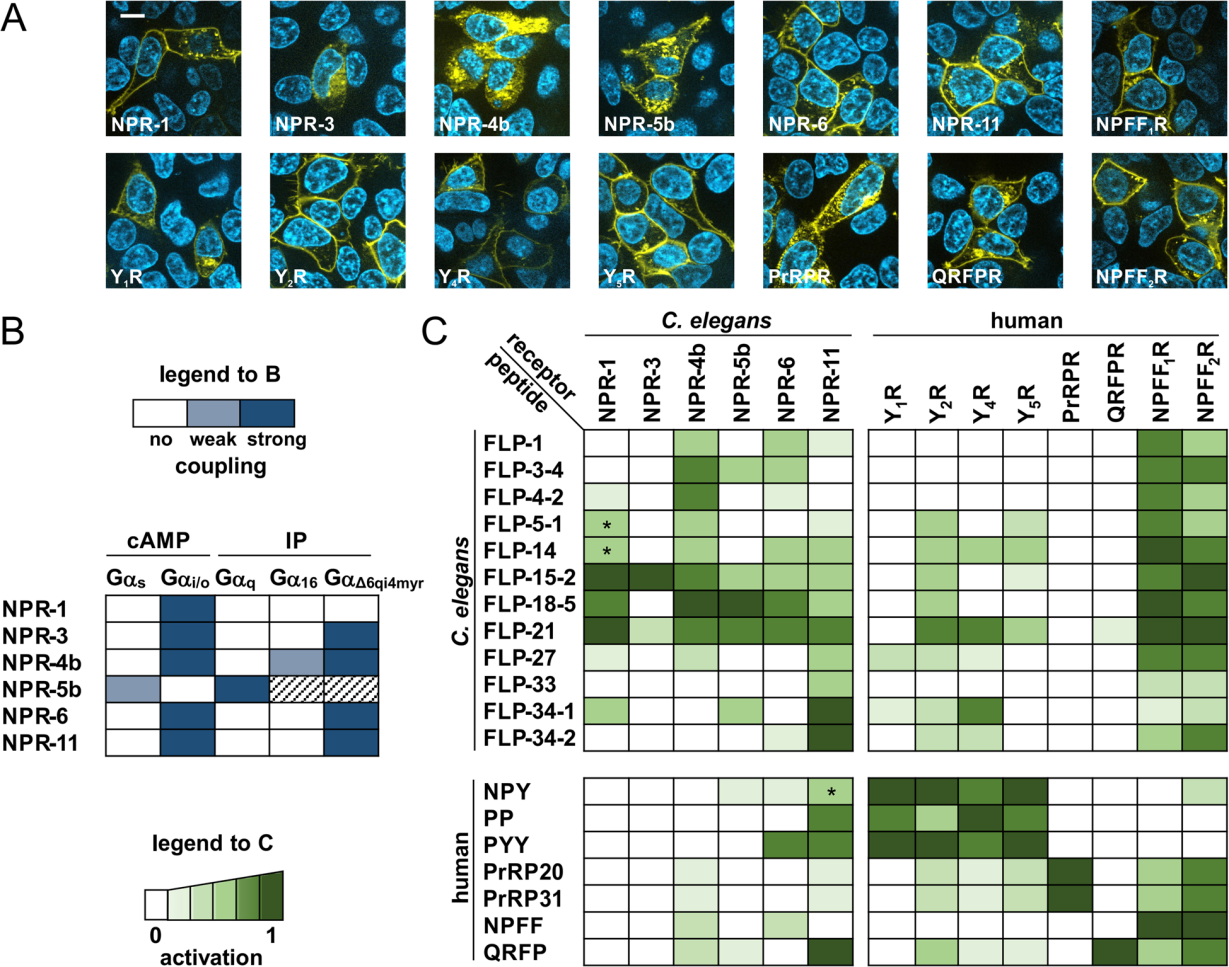
Basic pharmacological and cross-species reactivity studies of *C. elegans* and human neuropeptides and receptors in vitro. **a** Live-cell fluorescence microscopy of *C. elegans* NPR, human NPY and RFamide receptor::eYFP fusion proteins (yellow) shows similar receptor expression in transfected HEK293 cells and export to the plasma membrane. Images are representative of three independent experiments, and were acquired and processed identically. Nuclei were stained with Hoechst33342 (blue), scale bar = 10 μm. **b** *C. elegans* receptors recognize the human G protein repertoire and show distinct coupling preferences. Coupling specificity was deduced from second messenger production (cAMP or inositol phosphate (IP)) in response to FLP-5-1, FLP-15-2, FLP-18-5, and FLP-21, and qualitative data is depicted as color gradient (original numerical data are shown in Additional file 1: Tables S4 and S5). Coupling of NPR-5b to Gα_16_ and Gα_Δ6qi4myr_ via inositol phosphate accumulation is not readily accessible due to endogenous Gα_q_-coupling and was thus not determined (shaded). **c** Cross-species activation profiles of selected human and *C. elegans* receptors with different neuropeptides. Activation profiles were based on data from second messenger assays according to the endogenous G protein-coupling (original data can be found in Additional file 1: Tables S6–S9). Shown is a color-coded interaction matrix based on a two-concentration (0.1 μM; 10 μM) peptide screen as a coarse measure of peptide potency. The two lightest shades of green indicate partial receptor activation after stimulation with 10 μM peptide but no response to 0.1 μM of peptide (estimated EC_50_ > 10 μM assuming normal curve steepness and full agonism). Mid-grade colors display submaximal activation at 0.1 μM peptide and up to full activation at 10 μM (estimated EC_50_ 0.1 μM), while the darkest shade represents full activation in response to 0.1 μM of peptide (estimated EC_50_ < 100 nM). Few peptide/receptor combinations elicited identical, but submaximal responses at 0.1 and 10 μM concentration, indicative of partial agonism, and are marked with an asterisk (*). For more details on matrix generation in B and C, see Methods

All receptors were expressed heterologously in HEK293 cells and similar expression levels of human and *C. elegans* receptors as well as their presence in the plasma membrane was confirmed (Fig. 2a). One exception was *npr-3*, with weaker expression and only a fraction of the receptor being exported to the cell membrane.

We then characterized G protein-coupling abilities as these give first insights into their similarity to the human receptors, and orthologs are expected to have conserved G-protein preferences [28]. Functional studies of *C. elegans* receptors in human cell lines are possible due to the high conservation of G proteins across the animal kingdom [28] including complete identity in the α5 helix of the Gα subunit that mainly determines G-protein specificity [28–30]. G protein-coupling was identified using second messenger assays. Production of cAMP in the presence and absence of the adenylyl cyclase activator forskolin was employed to detect G_i/o_ and G_s_ activity, respectively, and accumulation of intracellular inositol phosphate (IP) concentrations served as indicator of G_q/11_ protein-coupling. As we did not observe any basal activity in the absence of agonistic peptides (Additional file 1: Table S8), we determined coupling preferences in response to the neuropeptides FLP-5-1, FLP-15-2, FLP-18-5, and FLP-21. Several studies individually tested some peptide-receptor combinations or their biological function (reviewed in [14]), so these peptides were chosen as ‘prototypic’ agonists to characterize or confirm the G protein-coupling of the selected receptors.

Activation of most *C. elegans* NPRs led to the inhibition of intracellular cAMP production, indicating coupling to G_i/o_ proteins (Additional file 1: Table S4). Only NPR-5b activation drove IP accumulation and a slight cAMP increase, suggesting coupling to G_q_ and G_s_ (Additional file 1: Tables S4 and S5). To establish a universal screening platform based on a robust signal accumulation, we co-transfected the *npr* constructs with plasmids encoding Gα_16_ or the chimeric Gα_Δ6qi4myr_ protein [20], which both mediate an increase in IP levels. Indeed, Gα_Δ6qi4myr_ efficiently yielded a signal for NPR-3, NPR-4b, NPR-6, and NPR-11 (Additional file 1: Table S5), while co-transfection of Gα_16_ exclusively rendered signaling of NPR-4b, albeit with lower signal windows. Interestingly, NPR-1 did not accept the Gα_Δ6qi4myr_ chimera, and activation was only monitored downstream of the endogenous Gα_i/o_ by decrease of cellular cAMP levels. G-protein preferences of all NPRs are summarized in Fig. 2b.

Next, we investigated activation of NPRs towards the entire panel of FLP ligands. We initially tested two peptide concentrations, 100 nM and 10 μM, which is in the range of reported EC_50_ values within the *C. elegans* NPR/FLP system (reviewed in [31, 32]) and likely at the upper limit of physiologically relevant concentrations (albeit local concentration in the synaptic cleft may be even up to mM range according to [33]), respectively. We found a high level of FLP promiscuity and consequently, redundancy in *C. elegans* receptor activation profiles (Fig. 2c). FLP-15-2 and FLP-21 activated all NPRs tested, while the remaining peptides stimulated at least three receptors. The more NPY-like peptides FLP-27, FLP-33, FLP-34-1, and FLP-34–2 appeared somewhat more selective, and mainly activated NPR-11 and NPR-1. Conversely, all NPRs were activated by at least five FLPs, albeit with different activities. One exception was NPR-3, which, in line with its weaker expression, displayed smaller signal windows and significant activation was only detected in response to FLP-15 and FLP-21.

For selected FLP-NPR pairs, we recorded full concentration-response curves to more accurately determine EC_50_ values (Additional file 1: Figure S1 and Table S11). As expected, all ligand-receptor interactions color-coded in dark green in Fig. 2 display an EC_50_ value below 100 nM. One of the most potent interactions was FLP-21 activating NPR-1 with a potency of 1 nM, in agreement with a previous study [31]. Interestingly, FLP-14 was a potent, but partial agonist at the NPR-1 (EC_50_ 24 nM, E_max_ 53% of FLP-21 induced response; Additional file 1: Figure S1). The RxRF/Ya-containing peptides FLP-34-1 (carrying a C-terminal tyrosine) and FLP-34–2 also reached nanomolar potencies at the NPR-11 receptor (EC_50_ FLP-34-1: 19 nM, FLP-34–2: 0.7 nM, Additional file 1: Figure S1, Table S11).

Next, we studied possible reactivity of *C. elegans* FLPs on human neuropeptide GPCRs and vice versa. We included NPY receptors, but also a group of related human RFamide receptors (PrRPR, QRFPR, and NPFFR). These display an equally high homology in pairwise alignments to NPRs and similar peptide ligands (Fig. 1), but have not yet been considered as potential orthologs since they were de-orphanized only after the seminal study on *npr-1* [12]. The ability of ligands to activate receptors of the other species can support an orthologous relationship as this usually goes along with pharmacological/functional conservation. However, the timewise evolutionary distance between *C. elegans* and human is large, reducing chances of cross-species reactivity. For this reason, we investigated all human NPY and RFamide receptors, including Y_4_R (and its ligand PP) and NPFF_2_R, which have evolved more recently during early vertebrate evolution [13, 34]. In this regard, we consider cross-species reactivity of a set of FLP-ligands (from local duplications during *C. elegans* evolution) to at least one receptor of a human receptor family (NPY or PrRPR or QRFPR or NPFFR) as functional similarity between these particular families, reflecting essential ligand requirements for receptor activation. Cross-species reactivity of an evolutionary ‘younger’ human peptide ligand to the ancient *C. elegans* NPR receptors might also occur, but is expected to be less likely. This is because a co-evolution of peptide and receptor typically creates more refined (and selective) ligand-receptor interactions over the long evolutional timescale. For instance, ligand length or surface electrostatics vary, which are key factors determining receptor-(subtype)-selectivity of peptide ligands [35, 36]. Thus, the ‘original’ binding pockets may be partially incompatible with or inaccessible for the resulting peptides.

Interestingly, human Y_2_, Y_4_ and Y_5_ receptors were activated by several *C. elegans* peptides including FLP-14 and FLP-21 and the RxRF/Ya containing peptides FLP-27, FLP-34-1, and FLP-34–2, while Y_1_R was only stimulated by FLP-27 and FLP-34-1. NPFF_1_R and NPFF_2_R were potently stimulated by almost all *C. elegans* peptides tested. Exceptions were FLP-34-1 and the human NPY/PP/PYY, which carry a C-terminal RY-amide indicating that the conserved RFamide motif plays a dominant role in receptor activation (in agreement with [37]). In contrast, the two human RFamide peptide receptors prolactin-releasing peptide receptor (PrRPR) and pyroglutamylated RFamide peptide receptor (QRFPR) did not respond to any *C. elegans* peptide (Fig. 2c, Additional file 1: Table S9).

Conversely, human neuropeptides also had activating abilities on *C. elegans*, albeit to a much more limited extent (Fig. 2c and Additional file 1: Table S6). NPR-11 displayed the most distinct activation by human NPY and RFa peptides, and we confirmed robust agonistic activity of PYY (EC_50_ 24 nM, Emax 86% of FLP-21-induced response) and partial agonism for NPY (EC_50_ 256 nM, E_max_ 49% of FLP-21-induced response) in full concentration-response curves (Additional file 1: Figure S1 and Table S11).

To check which residues are crucial for this cross-species activation, we applied peptides with the penultimate arginine exchanged for an alanine as this residue is essential for receptor-binding of human NPY and RFamide peptides [38, 39]. R8A control peptides of the universal ligands FLP-15-2 and FLP-21 drastically lost activity on all receptors (Additional file 1: Table S10 and Figure S1). Similarly, human [R35A]-PYY was inactive in two-point concentration screens and full concentration-response curves on *C. elegans* receptors NPR-6 and NPR-11 (Additional file 1: Table S10 and Figure S1), indicating that receptor-activation is mediated by the penultimate arginine residue of the peptide similar to the human system.

These data show that human NPY and NPFF and *C. elegans* FLP/NPR signaling systems display significant pharmacological overlap in activating preferentially the G_i/o_ pathway, cross-species activity of certain ligands and the requirement of the conserved penultimate arginine for receptor activation.

### Several human neuropeptide Y and FF receptors can phenotypically rescue physiological function in a *C. elegans npr-1* knockout strain

**Fig. 3:**
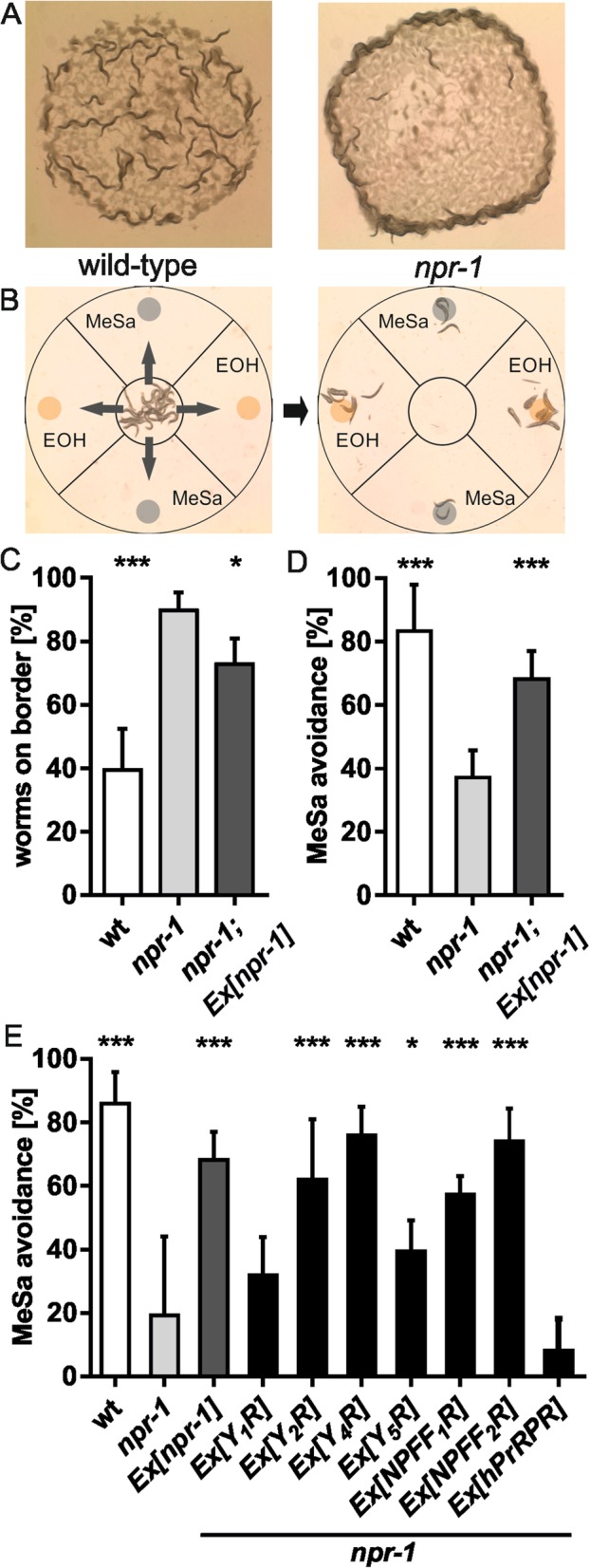
The effect of human neuropeptide receptors on bordering and MeSa avoidance behavior of *npr-1* null nematodes. **a** Assay layout for determining the bordering behavior of *C. elegans* nematodes. Adult hermaphrodites were transferred on a plate seeded with a defined bacterial lawn. Wild-type animals are dispersed randomly on the lawn (left) whereas *npr-1* null mutant worms form aggregates at the border of the bacterial lawn (right). **b** Schematic depiction of the assay set-up determining MeSa avoidance. Assay plates lacking bacteria are divided into four quadrants, containing a drop of ethanol (EOH) and sodium azide, or MeSa and sodium azide, respectively. Nematodes are initially placed in the middle of a plate (left). In the time-course of the assay, they crawl to the quadrants with MeSa or EOH and get paralyzed by the sodium azide (right). EOH serves as control substance, as it does not have any effect on *npr-1* mutant nematodes ([24] and Additional file 1: Figure S2). Worms in each quadrant are scored and MeSa avoidances is calculated. **c** The bordering phenotype of *npr-1* mutant individuals can be slightly ameliorated by transgenic expression of *npr-1* promoter-driven *npr-1*. Data are show as mean ± SD in at least four independent experiments (*n* ≥ 480). *p < 0.05; ***p < 0.001 compared to *npr-1* mutants. **d** MeSa avoidance of *npr-1* mutants is greatly reduced compared to wild-type animals. This phenotype is rescued by transgenic expression of *npr-1* driven by a *npr-1* promoter (*npr-1; Ex [npr-1]*). Data are shown as mean ± SD in at least six independent experiments (*n* ≥ 300). ***p < 0.001 compared to *npr-1* mutants. **e** Reduced MeSa avoidance of *npr-1* mutants is rescued by expression of several human neuropeptide receptors driven by a *npr-1* promoter from a transgene (*Ex [receptor]*). Construct *Ex [npr-1]* (also shown in (**d**)) served as a positive control*.* Data are shown as mean ± SD in at least six independent experiments (n ≥ 300). *p < 0.05; ***p < 0.001 compared to *npr-1* mutants

The potential of *C. elegans* and human ligands to cross-activate neuropeptide receptors of the other species in in vitro studies suggests that both neuropeptide systems share significant similarities and may adopt each other’s function. To elucidate this possibility, we used the neuropeptide receptor NPR-1 in *C. elegans* as a model. This GPCR has been reported to be activated by FLP-18 and FLP-21 [24, 31, 40], which is confirmed and extended by our study. As our data indicate that these neuropeptides are also capable to activate human Y_2_R, NPFF_1_R, and NPFF_2_R to elicit the same signal as NPR-1 (G_i/o_) (Fig. 2c), it is conceivable that these GPCRs can phenotypically rescue endogenous neuropeptide signaling in the *npr-1* knockout. Similar to members of the mammalian NPY receptor family, NPR-1 controls feeding, but also social behavior [12, 40–42]. Further functions of the receptor include response to heat [43] and substances such as ethanol [44] and methyl salicylate (MeSa) [24]. Consistently, a putative null mutation of the receptor, *npr-1(ky13)* [12], displays a multitude of phenotypic abnormalities, among others forming clumps and accumulating at the edge of the bacterial lawn as a result of social behavior [40] and reduced avoidance to MeSa [24]. These characteristics formed the basis for a read-out assay to test the ability of human neuropeptide GPCRs to functionally compensate loss of NPR-1. Two assays were established: one to measure accumulation of worms at the edge of the bacterial lawn (bordering) (Fig. 3a), the other to analyze avoidance to MeSa (Fig. 3b). As previously described [45], *npr-1(ky13)* mutants show a strong bordering phenotype (Fig. 3c) and a greatly reduced MeSa avoidance (Fig. 3d) compared to wild-type nematodes. An *npr-1(ky13)* strain expressing an *npr-1* promoter-driven *npr-1* from a transgene (construct was a kind gift of L. Ma) (Additional file 1: Figure S3A) rescued both phenotypes (Fig. 3c, d). However, the effect of the transgene to ameliorate the bordering phenotype was small, rendering this assay suboptimal. Thus, we focused on MeSa avoidance for testing heterologous neuropeptide GPCRs.

GFP-tagged human neuropeptide receptors Y_1_R, Y_2_R, Y_4_R, Y_5_R, NPFF_1_R, NPFF_2_R, and PrRPR were transgenically expressed under the control of the *npr-1* promoter in *npr-1(ky13)* nematodes (Additional file 1: Figure S3) and their capability to rescue the MeSa avoidance phenotype was analyzed (Fig. 3e). As hypothesized, Y_2_R, NPFF_1_R and NPFF_2_R were able to increase MeSa avoidance of *npr-1(ky13)* mutant nematodes to a similar level as transgenic *npr-1*. Interestingly, we observed that also Y_4_R and Y_5_R showed the ability to rescue the mutant phenotype, with Y_5_R to a lesser extent. Importantly, expression of Y_1_R or PrRPR did not rescue function, as expected from the lack of signaling response to any of the tested FLP in vitro*,* underlining the specificity of this rescue. These data show that the human neuropeptide receptors Y (Y_2_R, Y_4_R, Y_5_R) as well as FF (NPFF_1_R, NPFF_2_R) are able to phenotypically rescue the function of the *C. elegans* neuropeptide GPCR NPR-1.

### The FLP system acts context-specific with FLP-14 being essential for GPCR function in MeSa avoidance behavior

**Fig. 4:**
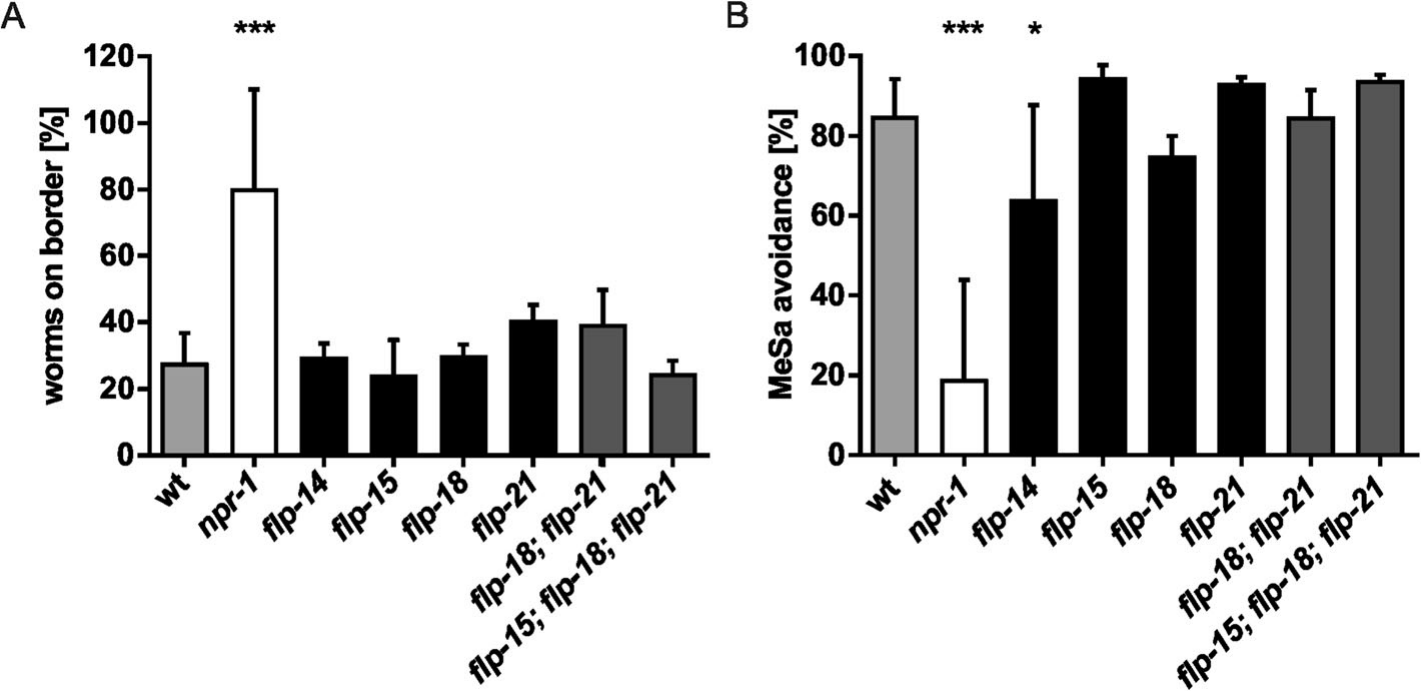
Loss-of-function of *C. elegans* FLP-14 phenocopies the *npr-1* null mutant in avoidance assay. Bordering behavior (**a**) and MeSa avoidance (**b**) of different *flp* loss-of-function mutants. *Flp-14* mutants show an effect in MeSa avoidance, but not in bordering behavior. Data are presented as mean ± SD in at least three independent experiments (*n* ≥ 150). *p < 0.05; ***p < 0.001 compared to wild-type

Given the multitude of FLPs activating NPR-1, as reported by us and others (reviewed in [14]), it is difficult to resolve which ligand(s) mediate specific functions in vivo*,* such as MeSa avoidance. We took advantage of the narrower pharmacological spectrum of human NPY receptors that were able to substitute NPR-1 function in vivo to investigate potential *flp* loss-of-function mutants. As the only neuropeptides that activate both NPR-1 and all GPCRs capable of rescuing its function are FLP-14 and FLP-21 (Fig. 2c), these were prime candidates. However, since NPR-1 has been described to bind to FLP-18 and FLP-21 [24, 31, 40] in various contexts and FLP-15 had the potential to strongly activate NPR-1 (Fig. 2c), we also tested strains deficient for these FLPs (*flp-14(gk3039), flp-15(gk1186), flp-18(gk3063)*, *flp-21(ok889)*) for their bordering and MeSa avoidance capabilities. Surprisingly, none of the mutants had any effect in the bordering assay (Fig. 4a). Likewise, neither *flp-18;flp-21* double mutants nor a *flp-15;flp-18;flp-21* triple mutant showed any phenotype similar to the one of *npr 1(ky13)* (Fig. 4a) suggesting the involvement of a different FLP and thus, a context-specific activation of NPR-1. Interestingly, *flp-14(gk3039)* mutants had a reduced MeSa avoidance while all other tested *flp* loss-of-function mutants were indistinguishable from wild-type (Fig. 4b), indicating an involvement of this so far nearly uncharacterized neuropeptide in chemosensation. As previous studies showed that *flp-18(gk3063)* mutants have a reduced MeSa avoidance [24], we analyzed *flp-18;flp-21* double and *flp-15;flp-18;flp-21* triple mutants to exclude any redundancies between these neuropeptides. However, none of them displayed any altered MeSa avoidance behavior.

Taken together, enabled by the pharmacological pattern of functional NPR-1 homologs from human we identified FLP-14 as a candidate for playing a role in avoidance of MeSa and interacting with NPR-1. Further, the FLP system seems to be highly context-specific.

## Discussion

Neuropeptidergic signaling is highly complex with many essential functions in mammals and deep evolutionary conservation. Global phylogenetic analyses [6, 7, 13] established a common neuropeptidergic signaling repertoire in the urbilaterian: About 30 different systems diversified prior to the split of protostomes and deuterostomes, including the NPF/Y, QRFPR, and NPFF families [6–8]. In the corresponding phylogenetic trees [6, 7], *C. elegans* NPRs are, however, placed next to the common bilaterian NPYR clade as representatives of a sNPF/FMRFa group, suggestive of a paralogous relationship to NPY receptors, but with a closer relationship to NPYR compared to other RFa receptors. However, reconstruction of vertebrate ancestral chromosomes [13] suggest that *C. elegans* NPRs have evolved from the most ancient NPY2/7R gene prior to further local gene duplications generating the NPY1/4/6R and NPY5R genes during early deuterostome evolution [13, 34] indicating that human NPYR and *C. elegans* NPRs are co-orthologs of the NPY2/7R ancestor. However, it cannot be ruled out that RFa receptors evolved later from a common ancestor in early deuterostome history followed by significant sequence divergence [13]. NPRs would then be expected to be equally distantly related to any of the human systems.

Here, we present the most comprehensive pharmacological and functional study of neuropeptidergic signaling in *C. elegans* to date gaining insights into relations between this system and the human NPY and RFamide systems PrRPR, QRFPR and NPFF_1/2_R, respectively.

Our data show functional similarities between *C. elegans* NPRs and the human NPY receptor system as well as NPFF receptors as human receptors recognize several FLPs and share the G_i/o_ protein preference with the *C. elegans* GPCRs. Further, human Y_2_R, Y_4_R as well as NPFF_1_R and NPFF_2_R rescue MeSa avoidance in *npr-1(ky13)* null mutants. It has to be noted that in contrast to other studies [46], bordering behavior is not rescued to a great extent by *npr-1*, which can be explained by the use of a different-sized *npr-1* promoter (2 kb versus > 3 kb).

Conserved G protein preference can be one indication for orthologous receptors [28]. Although G protein specificity might change during evolutionary timescales, this is not expected to be the case for all receptor subtypes. Mutations usually retain the ancient G-protein binding pocket and add new binding interfaces with distinct epitopes [28]. We note that we cannot fully exclude that NPRs also couple to other G proteins not detected in our assays despite decent expression levels and the fact that heterologous tissue culture systems have been proven valid for characterization of nematode GPCRs [47, 48]. As expected by a co-evolutionary scenario of the peptide-receptor interface outlined above, we find robust activation of human NPY and NPFF receptors by FLPs, but limited cross-species reactivity of human peptides at *C. elegans* NPRs. The most NPY-like FLPs FLP-27, FLP-33, FLP-34-1 and FLP-34–2 do not generally display higher potencies at the *C. elegans* or human receptors, but the second arginine apparently increases receptor specificity, also against the NPFF receptors.

Interestingly, PrRPR and QRFPR showed a distinct pharmacological profile: They were only activated by their endogenous ligands, and showed no phenotypic rescue in vivo. Moreover, these receptors couple to G_q_ proteins rather than G_i/o_ like most of the NPRs tested, arguing against an orthologous relationship.

Thus, our findings highlight functional similarities of *C. elegans* NPRs and human NPY receptors. Of note, we demonstrate for the first time that longer, more NPY-like peptides with a C-terminal RxRF/Ya sequence encoded by the *C. elegans* genome activate endogenous receptors, most prominently NPR-11 and NPR-1, and display cross-species activity at human NPY receptors. In this regard, also the presence of a C-terminal tyrosine (typical for vertebrate NPY-like peptides) in FLP-34-1 is tolerated for the activation of these *C. elegans* NPRs. This corresponds well to the recent suggestion that NPR-11 is the NPYR-ortholog based on sequence similarity to *Drosophila* NPFR and human NPY receptors [49], and is responsible for recognition of long NPY/NPF peptides. However, we find that NPR-11, but also the other NPRs were robustly activated by FLPs of FMRFa- as well as sNPF- and partially (long) NPF-type. Thus, our data challenge the concept of distinct *C. elegans* receptors for FMRFa, sNPF, and (long) NPF, respectively.

Surprisingly, functional similarities between NPR and NPFF receptors were found despite the suggestion that the latter form a more distant phylogenetic group orthologous to protostomian SIFamide receptors [6–8]. However, phylogenetic relationships in this case are dissimilar for receptors and ligands [7], with NPFFs forming a clade with protostomian FMRFa/sNPF peptides, consistent with our functional data. Likewise, similarities between NPFF and NPY receptors are supported by high sequence homology in humans [50] and shared ligand recognition via a conserved aspartate or glutamate at the top of transmembrane helix 6 (D/E6.59 Ballesteros and Weinstein nomenclature [51]) forming a salt bridge to the peptide’s penultimate arginine [39]. We identified this arginine, which is missing in SIFamides, also to be critical for FLPs to activate *C. elegans* and human receptors. NPR-11 does not display the typical acidic residue at position 6.59, but slightly displaced at E6.61, which is also enriched in acidic residues among NPY and RFa receptors. Similarities in peptide recognition are also underlined by the fact that residues E5.24 and Q3.32, which are important for binding of NPY and NPFF peptides [38, 52–54], are present in NPRs (Additional file 1: Figure S4), raising the question whether this is a case of convergent evolution or if orthologous relations need to be re-considered. Indeed, NPFF receptors appear quite permissive, in particular for short peptides. The only instances with negligible activity are NPY, PYY, PP and FLP-34-1, which carry a C-terminal RYamide. The phenylalanine to tyrosine exchange also reduces potency in the short endogenous NPFF ligand [37]. This lack of tolerance towards a C-terminal tyrosine is not seen in the FLP/NPR system, tentatively arguing against an orthologous relationship. In contrast, the interaction pattern of FLPs with NPYRs appeared more specific and a C-terminal tyrosine residue is accepted in the context of the FLP-34-1 peptide. In conjunction with the phylogenetic reconstructions and in vivo data, this more likely reflects an orthologous relationship. Due to our study design, we can, however, not rule out that the functional similarities between NPR and human NPY receptors, although striking, result from convergent evolution.

The comparative and comprehensive design of our study allowed to clarify several physiological aspects of *C. elegans* neuropeptide signaling and thus, highlights the value of such an approach. The activation pattern of NPRs displayed considerable redundancy as one receptor is activated by multiple FLPs. We suggest that this redundancy is a key in realizing context-specific receptor activation in distinct cells expressing distinct sets of FLPs. Context-dependent activation of one NPR by different FLPs might be one mechanism to orchestrate the diverse functions, among them roles in feeding and social behavior [12, 40–42], regulation of aerotaxis [45, 55, 56], response to ethanol [44], MeSa [24] and innate immunity [57, 58], all related to NPR-1. While NPR-1 is activated by FLP-21 to control social behavior [31, 45], we did not observe any effect of FLP-21 on MeSa avoidance indicating that this NPR-1 function is mediated by a different FLP. The combination of in vitro activation assays and rescue analyses using human GPCRs identified FLP-14 as a candidate for exerting the NPR-1 effect in response to MeSa. Interestingly, FLP-14 was a high-affinity partial agonist in our in vitro screening, which may contribute to a unique pharmacological profile. By maintaining a submaximal response, receptor desensitization (by arrestin-mediated or alternative mechanisms) is minimized, which helps to maintain the responsiveness of the addressed receptors and/or a certain ‘tone’ of an important biological signal. The role of this so far only poorly characterized FLP was confirmed by the *flp-14(gk3039)* null mutant, which phenocopies the MeSa avoidance seen in *npr-1(ky13)* null mutants. It needs to be noted that previous studies describe FLP-18 as an interactor of NPR-1 in MeSa avoidance [24]. Although a *flp-18(gk3063)* mutant shows a trend in avoidance behavior, we did not observe any significant effect, possibly originating from a slightly different assay set-up.

The concept of context-specific activation is supported by the distinct expression patterns of different *flps* (WormBase, release WS269): While FLP-14 is localized to the nerve ring, *flp-18* is expressed mainly in head, nerve ring and nerve cord neurons, whereas FLP-21 is present in neurons of the head, intestine and tale. There are only very few neurons in which overlapping expression of two of these neuropeptides has been described. Thus, context-specific expression of *flps* (and potentially their receptors) in combination with different specificities for NPRs might be one way of creating specificity in the highly complex FLP/NPR system. This somewhat resembles the situation in the human NPY system, in which the biological specificity also seems to arise from both, pharmacological (ligand) preferences and specific receptor expression and hormone secretion profiles. For instance, the selectivity between PP and NPY in activating the Y_4_R is backed up by the predominant expression of this receptor subtype in the gastrointestinal tract, where it senses comparably low levels of circulating of peptide hormones as part of the gut-brain axis [1, 4]. Moreover, the biological functions of the Y_1_ and Y_2_ receptors are divergent despite very similar functional affinities of NPY and PYY to activate the G_i_ pathway at these receptors [4]. Thus, the differential physiological functions must be encoded in the cellular expression in the brain, which is evidenced by expression studies (e.g. reviewed in [1]), and/or additional cellular pathways.

## Conclusions

In the present study, we provide evidence for a great extent of pharmacological and functional similarity between the *C. elegans* RFamide system (FLP/NPR) and human NPY as well as NPFF receptors. For the first time, we characterize NPY-like peptides (FLP-27, FLP-33, FLP-34-1, FLP-34–2) of *C. elegans* which underlines parallels in the peptide repertoire and requirements for receptor activation. This adds a functional perspective to current phylogenetic reconstructions and corroborates the suggested orthology between these systems. These findings demonstrate that they can serve as models to gain insights into the biology and molecular mechanisms of the respective other system. While several other organisms have been successfully employed to investigate functions of neuropeptides and their receptors such as *Drosophila melanogaster* (summarized in [10]), with the knowledge of our study on hand, the nematode offers distinct advantages for more generic studies. For instance, its transparency enables tracking of multiple fluorescently labeled peptides and monitoring their dynamics. This approach can also be conducted in a high-throughput manner, up to a 384-well format.

Thus, our study offers the foundation to investigate structure-function aspects of human receptors in an easily manageable in vivo model that we expect to be highly valuable for future studies.

## Supplementary information

**Additional file 1.** Supporting Results.

## Data Availability

This manuscript is accompanied by supporting information including peptide synthesis and analytical data; numerical data of the pharmacological analysis in vitro; oligonucleotides used for plasmid generation; *C. elegans* strains used in this study; expression control of human neuropeptide GPCRs in *C. elegans*; alignments of NPR receptors with human peptide-binding receptors. Further information and requests for resources and reagents should be directed to and will be fulfilled by Dr. Anette Kaiser and Dr. Simone Prömel.

## Abbreviations

(s)NPF: (Short) Neuropeptide F
ACN: Acetonitrile
*C. elegans*: *Caenorhabditis elegans*
cAMP: Cyclic adenosine monophosphate
CGC: *Caenorhabditis* Genetics Center
DMEM: Dulbecco’s modified Eagle’s medium
DMF: Dimethylformamide
EOH: Ethanol
eYFP: Enhanced yellow fluorescent protein
FCS: Fetal calf serum
FLP: FMRFamide-like peptide
Fmoc: Fluorenylmethyloxycarbonyl
FMRFa: Phe-Met-Arg-Phe-amide
GFP: Green fluorescent protein
GPCR: G protein-coupled receptor
IP: Inositol phosphate
MALDI-ToF: Matrix-assisted laser desorption/time of flight
MeSa: Methyl salicylate
NGM: Nematode growth medium
NPFF(R): Neuropeptide FF (receptor)
NPR: Neuropeptide receptor-resemblance
NPY: Neuropeptide Y
PLC: Phospholipase C
PP: Pancreatic polypeptide
PrRP(R): Prolactin-releasing peptide (receptor)
PYY: Peptide YY
QRFP(R): Pyroglutamylated RFamide peptide (receptor)
RP-HPLC: Reversed phase – high performance liquid chromatography
TFA: Trifluoroacetic acid
Y_x_R: NPY receptor subtype x

## References

[1] Yulyaningsih E, Zhang L, Herzog H, Sainsbury A (2011). NPY receptors as potential targets for anti-obesity drug development. Br J Pharmacol.

[2] Carvajal C, Dumont Y, Quirion R (2006). Neuropeptide y: role in emotion and alcohol dependence. CNS Neurol Disord Drug Targets.

[3] Thorsell A, Mathe AA (2017). Neuropeptide Y in alcohol addiction and affective disorders. Front Endocrinol (Lausanne).

[4] Pedragosa-Badia X, Stichel J, Beck-Sickinger AG (2013). Neuropeptide Y receptors: how to get subtype selectivity. Front Endocrinol (Lausanne).

[5] Lin S, Boey D, Herzog H (2004). NPY and Y receptors: lessons from transgenic and knockout models. Neuropeptides.

[6] Jekely G (2013). Global view of the evolution and diversity of metazoan neuropeptide signaling. Proc Natl Acad Sci U S A.

[7] Mirabeau O, Joly JS (2013). Molecular evolution of peptidergic signaling systems in bilaterians. Proc Natl Acad Sci U S A.

[8] Elphick Maurice R., Mirabeau Olivier, Larhammar Dan (2018). Correction: Evolution of neuropeptide signalling systems (doi:10.1242/jeb.151092). The Journal of Experimental Biology.

[9] Garczynski SF, Brown MR, Shen P, Murray TF, Crim JW (2002). Characterization of a functional neuropeptide F receptor from Drosophila melanogaster. Peptides.

[10] Bendena WG, Campbell J, Zara L, Tobe SS, Chin-Sang ID (2012). Select neuropeptides and their G-protein coupled receptors in Caenorhabditis Elegans and Drosophila melanogaster. Front Endocrinol (Lausanne).

[11] Nassel DR, Wegener C (2011). A comparative review of short and long neuropeptide F signaling in invertebrates: any similarities to vertebrate neuropeptide Y signaling?. Peptides.

[12] de Bono M, Bargmann CI (1998). Natural variation in a neuropeptide Y receptor homolog modifies social behavior and food response in C. elegans. Cell.

[13] Yun S, Furlong M, Sim M, Cho M, Park S, Cho EB, Reyes-Alcaraz A, Hwang JI, Kim J, Seong JY (2015). Prevertebrate local gene duplication facilitated expansion of the neuropeptide GPCR superfamily. Mol Biol Evol.

[14] Frooninckx L, Van Rompay L, Temmerman L, Van Sinay E, Beets I, Janssen T, Husson SJ, Schoofs L (2012). Neuropeptide GPCRs in *C. elegans*. Front Endocrinol (Lausanne).

[15] Husson SJ, Clynen E, Baggerman G, Janssen T, Schoofs L (2006). Defective processing of neuropeptide precursors in Caenorhabditis elegans lacking proprotein convertase 2 (KPC-2/EGL-3): mutant analysis by mass spectrometry. J Neurochem.

[16] Husson SJ, Schoofs L (2007). Altered neuropeptide profile of Caenorhabditis elegans lacking the chaperone protein 7B2 as analyzed by mass spectrometry. FEBS Lett.

[17] Brenner S (1974). The genetics of Caenorhabditis elegans. Genetics.

[18] Made V, Els-Heindl S, Beck-Sickinger AG (2014). Automated solid-phase peptide synthesis to obtain therapeutic peptides. Beilstein J Org Chem.

[19] Heckman KL, Pease LR (2007). Gene splicing and mutagenesis by PCR-driven overlap extension. Nat Protoc.

[20] Kostenis E (2002). Potentiation of GPCR-signaling via membrane targeting of G protein alpha subunits. J Recept Signal Transduct Res.

[21] Kostenis E, Waelbroeck M, Milligan G (2005). Techniques: promiscuous Galpha proteins in basic research and drug discovery. Trends Pharmacol Sci.

[22] Stinchcomb DT, Shaw JE, Carr SH, Hirsh D (1985). Extrachromosomal DNA transformation of Caenorhabditis elegans. Mol Cell Biol.

[23] Frokjaer-Jensen C, Davis MW, Hopkins CE, Newman BJ, Thummel JM, Olesen SP, Grunnet M, Jorgensen EM (2008). Single-copy insertion of transgenes in Caenorhabditis elegans. Nat Genet.

[24] Luo J, Xu Z, Tan Z, Zhang Z, Ma L (2015). Neuropeptide receptors NPR-1 and NPR-2 regulate Caenorhabditis elegans avoidance response to the plant stress hormone methyl salicylate. Genetics.

[25] Margie O, Palmer C, Chin-Sang I. *C. elegans* Chemotaxis Assay. J Vis Exp. 2013;(74):e50069. 10.3791/50069 PMC3667641 23644543

[26] Lesk AM. Introduction to bioinformatics. 1st ed. Oxford: Oxford University Press; 2002.

[27] Cardoso JC, Felix RC, Fonseca VG, Power DM (2012). Feeding and the rhodopsin family g-protein coupled receptors in nematodes and arthropods. Front Endocrinol (Lausanne).

[28] Flock T, Hauser AS, Lund N, Gloriam DE, Balaji S, Babu MM (2017). Selectivity determinants of GPCR-G-protein binding. Nature.

[29] Conklin BR, Farfel Z, Lustig KD, Julius D, Bourne HR (1993). Substitution of three amino acids switches receptor specificity of Gq alpha to that of Gi alpha. Nature.

[30] Komatsuzaki K, Murayama Y, Giambarella U, Ogata E, Seino S, Nishimoto I (1997). A novel system that reports the G-proteins linked to a given receptor: a study of type 3 somatostatin receptor. FEBS Lett.

[31] Kubiak TM, Larsen MJ, Nulf SC, Zantello MR, Burton KJ, Bowman JW, Modric T, Lowery DE (2003). Differential activation of “social” and “solitary” variants of the Caenorhabditis elegans G protein-coupled receptor NPR-1 by its cognate ligand AF9. J Biol Chem.

[32] Li C, Kim K (2014). Family of FLP peptides in Caenorhabditis elegans and related nematodes. Front Endocrinol (Lausanne).

[33] Mains RE, Eipper BA, Siegel GJ, Agranoff BW, Albers RW (1999). Neuropeptide receptors. In Basic Neurochemistry: Molecular, Cellular and Medical Aspects.

[34] Larhammar D, Salaneck E (2004). Molecular evolution of NPY receptor subtypes. Neuropeptides.

[35] Krumm BE, Grisshammer R (2015). Peptide ligand recognition by G protein-coupled receptors. Front Pharmacol.

[36] Joedicke L, Mao J, Kuenze G, Reinhart C, Kalavacherla T, Jonker HRA, Richter C, Schwalbe H, Meiler J, Preu J (2018). The molecular basis of subtype selectivity of human kinin G-protein-coupled receptors. Nat Chem Biol.

[37] Findeisen M, Rathmann D, Beck-Sickinger AG (2011). Structure-activity studies of RFamide peptides reveal subtype-selective activation of neuropeptide FF1 and FF2 receptors. Chem Med Chem.

[38] Merten N, Lindner D, Rabe N, Rompler H, Morl K, Schoneberg T, Beck-Sickinger AG (2007). Receptor subtype-specific docking of Asp6.59 with C-terminal arginine residues in Y receptor ligands. J Biol Chem.

[39] Findeisen M, Rathmann D, Beck-Sickinger AG (2011). RFamide peptides: structure, function, mechanisms and pharmaceutical potential. Pharmaceuticals.

[40] Rogers C, Reale V, Kim K, Chatwin H, Li C, Evans P, de Bono M (2003). Inhibition of Caenorhabditis elegans social feeding by FMRFamide-related peptide activation of NPR-1. Nat Neurosci.

[41] Gloria-Soria A, Azevedo RB (2008). npr-1 regulates foraging and dispersal strategies in Caenorhabditis elegans. Curr Biol.

[42] Milward K, Busch KE, Murphy RJ, de Bono M, Olofsson B (2011). Neuronal and molecular substrates for optimal foraging in Caenorhabditis elegans. Proc Natl Acad Sci U S A.

[43] Glauser DA, Chen WC, Agin R, Macinnis BL, Hellman AB, Garrity PA, Tan MW, Goodman MB (2011). Heat avoidance is regulated by transient receptor potential (TRP) channels and a neuropeptide signaling pathway in Caenorhabditis elegans. Genetics.

[44] Davies AG, Bettinger JC, Thiele TR, Judy ME, McIntire SL (2004). Natural variation in the npr-1 gene modifies ethanol responses of wild strains of C. elegans. Neuron.

[45] Rogers C, Persson A, Cheung B, de Bono M (2006). Behavioral motifs and neural pathways coordinating O2 responses and aggregation in C. elegans. Curr Biol.

[46] Macosko EZ, Pokala N, Feinberg EH, Chalasani SH, Butcher RA, Clardy J, Bargmann CI (2009). A hub-and-spoke circuit drives pheromone attraction and social behaviour in C. elegans. Nature.

[47] Müller A, Winkler J, Fiedler F, Sastradihardja T, Binder C, Schnabel R, Kungel J, Rothemund S, Hennig C, Schöneberg T, Prömel S (2015). Oriented Cell Division in the *C. elegans* Embryo Is Coordinated by G-Protein Signaling Dependent on the Adhesion GPCR LAT-1. PLoS Genet.

[48] Park YS, Cho TJ, Cho NJ (2006). Stimulation of cyclic AMP production by the Caenorhabditis elegans muscarinic acetylcholine receptor GAR-3 in Chinese hamster ovary cells. Arch Biochem Biophys.

[49] Fadda M, Hasakiogullari I, Temmerman L, Beets I, Zels S, Schoofs L (2019). Regulation of feeding and metabolism by neuropeptide F and short neuropeptide F in invertebrates. Front Endocrinol (Lausanne).

[50] Fredriksson R, Lagerstrom MC, Lundin LG, Schioth HB (2003). The G-protein-coupled receptors in the human genome form five main families. Phylogenetic analysis, paralogon groups, and fingerprints. Mol Pharmacol.

[51] Ballesteros JA, Weinstein H. Integrated methods for the construction of three-dimensional models and computational probing of structure-function relations in G protein-coupled receptors. In: Sealfon SC, editor. Methods in Neurosciences, vol. 25. Academic Press; 1995. p. 366–428.

[52] Kaiser A, Muller P, Zellmann T, Scheidt HA, Thomas L, Bosse M, Meier R, Meiler J, Huster D, Beck-Sickinger AG, Schmidt P (2015). Unwinding of the C-terminal residues of neuropeptide Y is critical for Y (2) receptor binding and activation. Angew Chem Int Ed Engl.

[53] Yang Z, Han S, Keller M, Kaiser A, Bender BJ, Bosse M, Burkert K, Kogler LM, Wifling D, Bernhardt G (2018). Structural basis of ligand binding modes at the neuropeptide Y Y1 receptor. Nature.

[54] Xu B, Vasile S, Ostergaard S, Paulsson JF, Pruner J, Aqvist J, Wulff BS, Gutierrez-de-Teran H, Larhammar D (2018). Elucidation of the binding mode of the Carboxyterminal region of peptide YY to the human Y2 receptor. Mol Pharmacol.

[55] Chang AJ, Chronis N, Karow DS, Marletta MA, Bargmann CI (2006). A distributed chemosensory circuit for oxygen preference in C. elegans. PLoS Biol.

[56] Cheung BH, Cohen M, Rogers C, Albayram O, de Bono M (2005). Experience-dependent modulation of C. elegans behavior by ambient oxygen. Curr Biol.

[57] Styer KL, Singh V, Macosko E, Steele SE, Bargmann CI, Aballay A (2008). Innate immunity in Caenorhabditis elegans is regulated by neurons expressing NPR-1/GPCR. Science.

[58] Nakad R, Snoek LB, Yang W, Ellendt S, Schneider F, Mohr TG, Rosingh L, Masche AC, Rosenstiel PC, Dierking K (2016). Contrasting invertebrate immune defense behaviors caused by a single gene, the Caenorhabditis elegans neuropeptide receptor gene npr-1. BMC Genomics.

