# Supplementary material for: Pharmacological and functional similarities of the human neuropeptide Y system in C. elegans challenges phylogenetic views on the FLP/NPR system
Source: Cell Commun Signal. 2019 Sep 18;17:123. doi: 10.1186/s12964-019-0436-1 (PMC6751662; doi:10.1186/s12964-019-0436-1)
Supplement: Supplementary file 1 — Additional file 1. Supporting Results. [file 12964_2019_436_MOESM1_ESM.docx]

**Additional file 1**

**Table S1. *C. elegans* strains used in this study.**

| **Strain** | **Genotype** | **Origin** | **Source** |
| --- | --- | --- | --- |
| N2 Bristol | wild-type | Brenner (1974) [1] | CGC |
| CX4148 | *npr-1(ky13) X* | de Bono and Bargmann (1998) [2] | CGC |
| RB982 | *flp-21(ok889) V* | *C. elegans* Gene Knockout Consortium | CGC |
| VC2504 | *flp-15(gk1186) III* | *C. elegans* Gene Knockout Consortium | CGC |
| APR544 | *flp-21(ok889) V; flp-18(gk3063) X* | this study | --- |
| APR560 | *flp-15 (gk1186) III; flp-21(ok889) V; flp-18(gk3063) X* | this study | --- |
| APR571, APR572 | *npr-1(ky13) X; aprEx226 [pSP137, pmyo-2::mCherry, pBSK]* | this study | --- |
| APR573 | VC2016, *flp-18(gk3063) X,* 2x outcrossed with N2 | this study | --- |
| APR574 | VC1957, *flp-14(gk3039) III*, 2x outcrossed with N2 | this study | --- |
| APR575, APR576 | *npr-1(ky13) X; aprEx228-229 [npr-1p::npr-1::gfp, pmyo-3::mCherry, pBSK]* | this study | --- |
| APR577,  APR578 | *npr-1(ky13) X; aprEx230-231 [pSP141, pmyo-3::mCherry, pBSK]* | this study | --- |
| APR579, APR580 | *npr-1(ky13) X; aprEx232-233 [pSP136, pmyo-3::mCherry, pBSK]* | this study | --- |
| APR581 | *npr-1(ky13) X; aprEx234 [pSP138, pmyo-3::mCherry, pBSK]* | this study | --- |
| APR582, APR583 | *npr-1(ky13) X; aprEx235-236 [pSP139, pmyo-3::mCherry, pBSK]* | this study | --- |
| APR584, APR585 | *npr-1(ky13) X; aprEx237-238 [pSP133, pmyo-2::mCherry, pBSK]* | this study | --- |
| APR586, APR587 | *npr-1(ky13) X; aprEx239-240 [pSP148, pmyo-3::mCherry, pBSK]* | this study | --- |

**Table S2. Sequence of primers used to generate constructs presented in the study.**

| **Primer** | **Sequence (5’- 3’)** |
| --- | --- |
| NPR-1-MluI-f | AAAACGCGTGCCACCATGGAAGTTGAAAATTTTACCGAC |
| NPR-1-Linker-r | GCTCACGACCGGTGGATCGACTAGCGTGTCGTTGACG |
| NPR-3-MluI-f | AAAACGCGTGCCACCATGGAGGGTGGTCGAAACTG |
| NPR-3-Linker-r | GCTCACGACCGGTGGATCTAAAAGTTGATCTCCAGCTCG |
| NPR-4b-MluI-f | AAAACGCGTGCCACCATGTTACTGGAAATTGGCAC |
| NPR-4b-Linker-r | GCTCACGACCGGTGGATCGAAAGAAGCCTTCCTTGGTAG |
| NPR-5b-MluI-f | AAAACGCGTGCCACCATGGTTAGTTCGGCGGCC |
| NPR-5b-Linker-r | GCTCACGACCGGTGGATCCAGCATCTCGCGGGTAG |
| NPR-6-MluI-f | AAAACGCGTGCCACCATGTCGAATGATCTCGTGCC |
| NPR-6-Linker-r | GCTCACGACCGGTGGATCAAGCAGAAGCGTCTGACAAC |
| NPR-11-MluI-f | AAAACGCGTGCCACCATGGGATCGGTGAATGAATC |
| NPR-11-Linker-r | GCTCACGACCGGTGGATCCAGCTGCTCATCTTGCTCC |
| Linker-YFP-f | GATCCACCGGTCGTGAGC |
| YFP-XbaI-NheI-r | TTTGCTAGCGTGTTACCCCTCTAGACCTG |
| pPD95.79_f | ACCGGTAGAAAAAATGAGTA |
| pPD95.79_r | CCCGGGGATCCTCTAGAGTC |
| npr-1p_f | CCCGGGtgttagtcaattaccatac |
| npr-1p_r | cccgggttggcctatgtctgaaattt |
| npy1R_f | ACCGGTATGAATTCAACATTATTTTCC |
| npy1R_r | ACCGGTGAGATTTTTTCATTATC |
| npy2R_f | ACCGGTATGGGTCCAATAGGTGCAGAGGCT |
| npy2R_r | ACCGGTTGGACATTGGTAGCCTCTG |
| npy4R_f | ACCGGTATGAACACCTCTCACCTCCT |
| npy4R_r | ACCGGTACAATGGGATTGGACCT |
| npy5R_f | ACCGGTATGGATTTAGAGCTCGACGA |
| npy5R_r | ACCGGTTCCATATGAAGACAGTGTATAAGG |
| npffr-1_f | ACCGGTATGGAGGGGGAGCCCTCC |
| npffr-1_f | ACCGGTCCGATATCCCAGGCTGGAA |
| npffr-2_f | ACCGGTATGAATGAGAAATGGGACAC |
| npffr-2_r | ACCGGTACAATCTCACTGCTGTTAG |
| prlhr_f | ACCGGTATGGCCTCATCGACCACTC |
| prlhr_r | ACCGGTGCGATGACCACGCTGACGG |

**Table S3. Analytical data of synthesized peptides.** RP-HPLC solvents: A = H_2_O + 0.1 % TFA; B = acetonitrile (ACN) + 0.08% TFA. For peptides marked with ($) solvents: A = H_2_O + 10 mM (NH_4_)_2_CO_2_, pH 8.5; B = 80% ACN + 10 mM (NH_4_)_2_CO_2_, pH 8.5.

| **Peptide** | **Sequence** | **Purity [%] †** | **Elution [%B] †** | **MALDI-ToF MS** | |
| --- | --- | --- | --- | --- | --- |
|  |  |  |  | **M_calc_ [Da]** | **[M+H]^+^ [Da]** |
| FLP-1-2 | AGSDPNFLRF-NH2 | >95%^1,3,a^ | 42.8^1,a^ | 1121.5 | 1122.5 |
| FLP-3-4 ^$^ | NPENDTPFGTMRF-NH2 | >95%^1,3,a^ | 39.2^1,a^ | 1523.6 | 1524.6 |
| FLP-4-2 | ASPSFIRF-NH2 | >95%^1,3,a^ | 42.3^1,a^ | 922.5 | 923.5 |
| FLP-5-1 | APKPKFIRF-NH2 | >95%^1,3,a^ | 38.8^1,a^ | 1101.7 | 1102.7 |
| FLP-14 | KHEYLRF-NH2 | >95%^1,3,a^ | 37.8^1,a^ | 990.5 | 991.5 |
| FLP-15-2 ^$^ | RGPSGPLRF-NH2 | >95%^1,3,a^ | 38.5^1,a^ | 984.5 | 985.5 |
| [R8A]FLP-15-2 | RGPSGPLAF-NH2 | >95%^1,2,a^ | 41.0^1,a^ | 899.5 | 900.5 |
| FLP-18-5 ^$^ | SEVPGVLRF-NH2 | >95%^1,3,a^ | 44.8^1,a^ | 1001.5 | 1002.5 |
| FLP-21 ^$^ | GLGPRPLRF-NH2 | >95%^1,3,a^ | 39.3^1,a^ | 1010.6 | 1011.6 |
| [R8A]FLP-21 | GLGPRPLAF-NH2 | >95%^1,2,a^ | 42.3^1,a^ | 925.6 | 926.6 |
| FLP-27 | EASAFGDIIGELKGKGLGGRMRF-NH2 | >95%^1,3,a^ | 44.8^1,a^ | 2407.3 | 2408.3 |
| FLP-33 | APLEGFEDMSGFLRTIDGIQKPRF-NH2 | >95%^1,3,a^ | 49.1^1,a^ | 2722.4 | 2722.4 |
| FLP-34-1 | ADISTFASAINNAGRLRY-NH2 | >90%^1,3,a^ | 43.6^1,a^ | 1938.0 | 1939.0 |
| FLP-34-2 | ALNRDSLVASLNNAERLRF-NH2 | >95%^1,2,a^ | 43.3^1,a^ | 2157.2 | 2158.2 |
| NPY | YPSKPDNPGEDAPAEDLARYYSALRHYINLITRQRY-NH2 | >95%^4,a,b^ | 39.8^4,a^ | 4251.1 | 4252.1 |
| PP | APLEPVYPGDNATPEQMAQYAADLRRYINMLTRPRY-NH2 | >95%^4,a,b^ | 41.4^4,a^ | 4179.1 | 4180.1 |
| PYY | YPIKPEAPGEDASPEELNRYYASLRHYLNLVTRQRY-NH2 | >94%^4,a,b^ | 36.1^4,a^ | 4307.2 | 4308.2 |
| [R35A]PYY | YPIKPEAPGEDASPEELNRYYASLRHYLNLVTRQAY-NH2 | >95%^1,3,a^ | 46.6^1,a^ | 4222.1 | 4223.1 |
| PrRP20 | TPDINPAWYASRGIRPVGRF-NH2 | >95%^1,2,a^ | 41.2^1,a^ | 2271.2 | 2272.2 |
| PrRP31 | SRTHRHSMEIRTPDINPAWYASRGIRPVGRF-NH2 | >95%^1,3,a^ | 40.5^1,a^ | 3661.9 | 3662.9 |
| NPFF | FLFQPQRF-NH2 | >95%^1,3,a^ | 44.5^1,a^ | 1080.6 | 1081.6 |
| QRFP26 | TSGPLGNLAEELNGYSRKKGGFSFRF-NH2 | >95%^1,3,a^ | 45.2^1,a^ | 2830.5 | 2831.5 |

**†** Gradients: (1) 10–60% B in 30 min, (2) 20–60% B in 40 min, (3) 20–60% B in 30 min, (4) 20–70% B in 40 min; RP‐HPLC columns: (a) Phenomenex Jupiter C-12, 90 Å, (b) Phenomenex Aeris XB‐C18, 100 Å.

**Table S4. Profiling of G protein-coupling specificity – cAMP assay data** **of *C. elegans* NPRs.** Coupling to G_s_ and G_i/o_ was assessed via a cAMP reporter gene assay in transiently transfected HEK293 cells. For measuring G_i/o_ activity, the inhibition of cAMP production induced by 1 µM forskolin (set to 1.00) was measured, i.e. lower values correspond to high G_i/o_ activity. Receptor activation by a peptide is highlighted in bold (cAMP inhibition > 20% or cAMP stimulation > 2-fold, respectively, and statistically significant from buffer control tested by two-way ANOVA with Bonferroni's post-hoc test with p values ≤ 0.05). Data are shown as x-fold of forskolin (mean ± SEM) of at least three independent experiments.

|  |  |  |  | **NPR-1** | | **NPR-3** | | **NPR-4b** | | **NPR-5b** | | **NPR-6** | | **NPR-11** | |
| --- | --- | --- | --- | --- | --- | --- | --- | --- | --- | --- | --- | --- | --- | --- | --- |
|  |  |  |  | mean | ±SEM | mean | ±SEM | mean | ±SEM | mean | ±SEM | mean | ±SEM | mean | ±SEM |
| **cAMP assay** | **G_s_ [x-fold of basal]** | **w/o** | / | 0.94 | 0.06 | 1.00 | 0.06 | 1.00 | 0.03 | 1.00 | 0.02 | 1.00 | 0.03 | 1.00 | 0.04 |
|  |  | **FLP-5-1** | 0.1 µM | 1.12 | 0.07 | 0.86 | 0.04 | 0.89 | 0.04 | 0.68 | 0.07 | 0.68 | 0.07 | 0.70 | 0.06 |
|  |  |  | 10 µM | 1.05 | 0.09 | 0.85 | 0.11 | 0.92 | 0.09 | 0.68 | 0.07 | 0.57 | 0.05 | 0.69 | 0.06 |
|  |  | **FLP-15-2** | 0.1 µM | 1.01 | 0.09 | 0.99 | 0.18 | 0.96 | 0.10 | 0.83 | 0.11 | 0.62 | 0.07 | 0.70 | 0.05 |
|  |  |  | 10 µM | 1.01 | 0.09 | 0.96 | 0.15 | 0.98 | 0.08 | 1.03 | 0.17 | 0.56 | 0.07 | 0.69 | 0.05 |
|  |  | **FLP-18-5** | 0.1 µM | 0.90 | 0.03 | 0.95 | 0.07 | 0.90 | 0.04 | **2.04** | **0.41** | 0.85 | 0.04 | 0.93 | 0.09 |
|  |  |  | 10 µM | 0.95 | 0.06 | 0.74 | 0.04 | 0.83 | 0.03 | **2.14** | **0.31** | 0.86 | 0.05 | 0.92 | 0.08 |
|  |  | **FLP-21** | 0.1 µM | 1.01 | 0.06 | 0.91 | 0.05 | 0.87 | 0.04 | 1.71 | 0.29 | 0.89 | 0.04 | 1.16 | 0.13 |
|  |  |  | 10 µM | 0.94 | 0.07 | 0.77 | 0.03 | 0.93 | 0.05 | 1.59 | 0.36 | 0.58 | 0.04 | 0.68 | 0.04 |
|  |  | **forskolin** | 1 µM | 15.39 | 5.61 | 20.07 | 9.11 | 16.52 | 4.40 | 23.17 | 9.99 | 12.19 | 4.31 | 16.67 | 3.12 |
|  |  |  | 100 µM | 76.79 | 4.28 | 51.90 | 3.40 | 44.21 | 2.12 | 67.99 | 3.67 | 46.24 | 4.98 | 60.68 | 6.42 |
|  | **G_i_ [x-fold of forskolin]** | **w/o** | / | 0.07 | 0.01 | 0.06 | 0.01 | 0.07 | 0.01 | 0.05 | 0.01 | 0.09 | 0.01 | 0.06 | 0.00 |
|  |  | **forskolin** | 1 µM | 1.00 | 0.07 | 1.00 | 0.10 | 1.00 | 0.02 | 1.00 | 0.10 | 1.00 | 0.04 | 1.00 | 0.05 |
|  |  | **FLP-5-1** | 0.1 µM | **0.58** | **0.05** | 0.68 | 0.07 | **0.75** | **0.04** | 0.92 | 0.08 | 0.85 | 0.08 | **0.94** | **0.07** |
|  |  |  | 10 µM | **0.53** | **0.05** | 0.87 | 0.05 | **0.40** | **0.03** | 1.90 | 0.20 | 1.01 | 0.06 | **0.51** | **0.05** |
|  |  | **FLP-15-2** | 0.1 µM | **0.26** | **0.03** | **0.29** | **0.03** | **0.50** | **0.02** | **1.07** | **0.09** | **0.75** | **0.04** | **0.88** | **0.05** |
|  |  |  | 10 µM | **0.18** | **0.01** | **0.14** | **0.01** | **0.24** | **0.01** | **4.21** | **0.36** | **0.26** | **0.02** | **0.35** | **0.03** |
|  |  | **FLP-18-5** | 0.1 µM | **0.68** | **0.09** | 0.84 | 0.07 | **0.23** | **0.01** | **4.76** | **0.46** | **0.73** | **0.04** | **0.71** | **0.09** |
|  |  |  | 10 µM | **0.42** | **0.05** | 0.98 | 0.08 | **0.23** | **0.03** | **5.94** | **0.49** | **0.29** | **0.03** | **0.70** | **0.05** |
|  |  | **FLP-21** | 0.1 µM | **0.20** | **0.02** | **0.86** | **0.09** | **0.65** | **0.04** | **2.66** | **0.25** | **0.62** | **0.04** | **0.35** | **0.02** |
|  |  |  | 10 µM | **0.22** | **0.02** | **0.59** | **0.04** | **0.32** | **0.02** | **5.84** | **0.53** | **0.26** | **0.03** | **0.21** | **0.02** |

**Table S5. Profiling of G protein-coupling specificity – inositol phosphate (IP) accumulation data of *C. elegans* NPRs.** Coupling to Gα_q_, promiscuous Gα_16_ and chimera Gα_Δ6qi4myr_ was observed by inositol phosphate accumulation in transiently transfected HEK293 cells. For testing Gα_16_- and Gα_Δ6qi4myr_-coupling, the corresponding Gα protein instead of empty vector (mock) was co-transfected. Receptor activation by a peptide is highlighted in bold (signal > 2-fold and statistically significant from basal as tested by two way ANOVA with Bonferroni's post-hoc test and p values ≤ 0.05). Measuring coupling of NPR-5b to Gα_16_ and Gα_Δ6qi4myr_ via inositol phosphate accumulation is not readily accessible due to endogenous Gα_q_-coupling and is thus marked as not determinable (n.d.). Receptors are arranged horizontally, peptides vertically. Data represent x‑fold of basal (mean ± SEM) of at least three independent experiments.

|  |  |  |  | **NPR-1** | | **NPR-3** | | **NPR-4b** | | **NPR-5b** | | **NPR-6** | | **NPR-11** | |
| --- | --- | --- | --- | --- | --- | --- | --- | --- | --- | --- | --- | --- | --- | --- | --- |
|  |  |  |  | mean | ±SEM | mean | ±SEM | mean | ±SEM | mean | ±SEM | mean | ±SEM | mean | ±SEM |
| **IP assay** | **endog. Gα_q_ + mock [x-fold of basal]** | **w/o** | / | 1.00 | 0.12 | 1.00 | 0.06 | 1.00 | 0.10 | 1.00 | 0.03 | 1.00 | 0.04 | 1.00 | 0.05 |
|  |  | **FLP-5-1** | 0.1 nM | 0.89 | 0.10 | 0.92 | 0.07 | 0.98 | 0.08 | 0.93 | 0.05 | 0.92 | 0.05 | 1.05 | 0.09 |
|  |  |  | 10 µM | 0.94 | 0.12 | 1.07 | 0.09 | 0.99 | 0.05 | 1.11 | 0.05 | 0.95 | 0.04 | 1.09 | 0.12 |
|  |  | **FLP-15-2** | 0.1 nM | 0.98 | 0.10 | 0.92 | 0.05 | 0.85 | 0.08 | **1.07** | **0.05** | 0.93 | 0.07 | 1.02 | 0.08 |
|  |  |  | 10 µM | 1.00 | 0.12 | 0.92 | 0.05 | 1.14 | 0.12 | **3.50** | **0.64** | 1.02 | 0.10 | 1.03 | 0.05 |
|  |  | **FLP-18-5** | 0.1 nM | 0.89 | 0.09 | 0.99 | 0.06 | 1.13 | 0.15 | **4.81** | **0.91** | 1.03 | 0.06 | 1.10 | 0.10 |
|  |  |  | 10 µM | 0.91 | 0.10 | 0.95 | 0.06 | 1.09 | 0.11 | **15.47** | **2.96** | 1.06 | 0.11 | 0.95 | 0.06 |
|  |  | **FLP-21** | 0.1 nM | 1.03 | 0.13 | 1.11 | 0.07 | 0.91 | 0.06 | **1.49** | **0.17** | 0.96 | 0.03 | 0.99 | 0.12 |
|  |  |  | 10 µM | 0.93 | 0.09 | 0.94 | 0.09 | 1.01 | 0.07 | **13.94** | **2.87** | 0.97 | 0.16 | 1.13 | 0.13 |
|  | **endog. Gα_q_ + Gα_16_ [x-fold of basal]** | **w/o** | / | 1.00 | 0.10 | 1.00 | 0.10 | 1.00 | 0.06 | n.d. | | 1.00 | 0.08 | 1.00 | 0.11 |
|  |  | **FLP-5-1** | 0.1 nM | 0.90 | 0.10 | 0.93 | 0.08 | 1.04 | 0.08 |  |  | 1.01 | 0.12 | 1.04 | 0.14 |
|  |  |  | 10 µM | 0.81 | 0.07 | 0.81 | 0.05 | 0.84 | 0.08 |  |  | 0.96 | 0.09 | 0.84 | 0.08 |
|  |  | **FLP-15-2** | 0.1 nM | 1.01 | 0.09 | 1.09 | 0.15 | 1.11 | 0.14 |  |  | 0.99 | 0.11 | 1.00 | 0.11 |
|  |  |  | 10 µM | 0.84 | 0.08 | 0.79 | 0.06 | 0.99 | 0.10 |  |  | 0.96 | 0.10 | 0.97 | 0.10 |
|  |  | **FLP-18-5** | 0.1 nM | 0.98 | 0.12 | 0.96 | 0.09 | **1.05** | **0.08** |  |  | 0.91 | 0.05 | 1.11 | 0.11 |
|  |  |  | 10 µM | 0.90 | 0.07 | 0.97 | 0.11 | **2.71** | **0.25** |  |  | 1.03 | 0.06 | 0.91 | 0.08 |
|  |  | **FLP-21** | 0.1 nM | 1.06 | 0.11 | 0.94 | 0.11 | 0.96 | 0.11 |  |  | 0.99 | 0.10 | 0.94 | 0.12 |
|  |  |  | 10 µM | 0.90 | 0.07 | 0.85 | 0.07 | 0.91 | 0.12 |  |  | 1.13 | 0.13 | 2.05 | 0.26 |
|  | **endog. Gα_q_ + Gα_Δ6qi4myr_ [x-fold of basal]** | **w/o** | / | 1.00 | 0.09 | 1.00 | 0.13 | 1.00 | 0.13 | n.d. | | 1.00 | 0.12 | 1.00 | 0.11 |
|  |  | **FLP-5-1** | 0.1 nM | 0.88 | 0.08 | 0.88 | 0.07 | **2.00** | **0.81** |  |  | 0.96 | 0.12 | **1.03** | **0.05** |
|  |  |  | 10 µM | 0.93 | 0.10 | 0.95 | 0.08 | **5.53** | **0.68** |  |  | 0.98 | 0.13 | **6.49** | **0.43** |
|  |  | **FLP-15-2** | 0.1 nM | 0.94 | 0.08 | **1.40** | **0.16** | **4.58** | **0.40** |  |  | **1.02** | **0.15** | **1.19** | **0.08** |
|  |  |  | 10 µM | 1.01 | 0.07 | **2.31** | **0.26** | **12.19** | **0.89** |  |  | **7.37** | **0.57** | **12.88** | **1.21** |
|  |  | **FLP-18-5** | 0.1 nM | 0.87 | 0.07 | 0.91 | 0.12 | **11.51** | **0.97** |  |  | **1.26** | **0.16** | **0.81** | **0.04** |
|  |  |  | 10 µM | 0.87 | 0.09 | 1.00 | 0.05 | **14.41** | **1.01** |  |  | **8.17** | **0.59** | **3.73** | **0.34** |
|  |  | **FLP-21** | 0.1 nM | 0.99 | 0.14 | 0.95 | 0.07 | **3.22** | **0.34** |  |  | **1.47** | **0.20** | **7.35** | **0.54** |
|  |  |  | 10 µM | 0.90 | 0.07 | 0.96 | 0.08 | **10.86** | **0.93** |  |  | **9.35** | **1.00** | **20.41** | **1.75** |

**Table S6. Cross-species reactivity - cAMP assay data of *C. elegans* receptors.** A cAMP reporter gene assay was used to assess coupling to G_i/o_ in transiently transfected HEK293 cells. G_i/o_ activity was measured by the inhibition of cAMP production induced by 1 µM forskolin (set to 1.00), i.e. lower values correspond to high G_i/o_ activity. Receptor activation by a peptide is highlighted in bold (cAMP inhibition > 20% or cAMP stimulation > 2-fold, respectively, and statistically significant from buffer control as tested by one way ANOVA with Dunnett's post-hoc test and p values ≤ 0.05). Peptides that elicit an equal, but submaximal signal at 0.1 and 10 µM concentration at a given receptor indicative of partial agonism are marked with an asterisk (*). Data represent x‑fold of forskolin (mean ± SEM) of at least three independent experiments.

|  |  |  | **NPR-1** | | **NPR-3** | | **NPR-4b** | | **NPR-5b** | | **NPR-6** | | **NPR-11** | |
| --- | --- | --- | --- | --- | --- | --- | --- | --- | --- | --- | --- | --- | --- | --- |
|  |  |  | mean | ±SEM | mean | ±SEM | mean | ±SEM | mean | ±SEM | mean | ±SEM | mean | ±SEM |
| ***C. elegans*** | **FLP-1** | 0.1 µM | 0.98 | 0.10 | 0.87 | 0.08 | **0.81** | **0.04** | 1.02 | 0.10 | **0.94** | **0.10** | **0.92** | **0.06** |
|  |  | 10 µM | 0.89 | 0.07 | 1.02 | 0.08 | **0.42** | **0.03** | 1.72 | 0.14 | **0.29** | **0.02** | **0.72** | **0.05** |
|  | **FLP-3-4** | 0.1 µM | 0.87 | 0.07 | 0.85 | 0.07 | **0.67** | **0.04** | **1.10** | **0.07** | **0.85** | **0.06** | 0.80 | 0.06 |
|  |  | 10 µM | 0.89 | 0.07 | 0.89 | 0.08 | **0.23** | **0.02** | **3.95** | **0.40** | **0.26** | **0.03** | 0.97 | 0.05 |
|  | **FLP-4-2** | 0.1 µM | **0.77** | **0.10** | 0.94 | 0.11 | **0.52** | **0.04** | 0.90 | 0.08 | **0.86** | **0.08** | 0.89 | 0.06 |
|  |  | 10 µM | **0.76** | **0.05** | 0.83 | 0.07 | **0.19** | **0.01** | 1.82 | 0.10 | **0.72** | **0.04** | 0.83 | 0.06 |
|  | **FLP-5-1** | 0.1 µM | **0.54*** | **0.06** | 0.66 | 0.07 | **0.73** | **0.04** | 0.91 | 0.08 | 0.84 | 0.09 | **0.94** | **0.08** |
|  |  | 10 µM | **0.50*** | **0.05** | 0.86 | 0.06 | **0.36** | **0.03** | 1.96 | 0.22 | 1.01 | 0.07 | **0.48** | **0.05** |
|  | **FLP-14** | 0.1 µM | **0.66*** | **0.05** | 0.76 | 0.04 | **0.74** | **0.03** | 0.89 | 0.09 | **0.87** | **0.07** | **0.73** | **0.09** |
|  |  | 10 µM | **0.62*** | **0.07** | 0.80 | 0.05 | **0.37** | **0.03** | 1.43 | 0.31 | **0.17** | **0.01** | **0.31** | **0.03** |
|  | **FLP-15-2** | 0.1 µM | **0.20** | **0.03** | **0.25** | **0.03** | **0.47** | **0.02** | **1.07** | **0.10** | **0.72** | **0.04** | **0.87** | **0.05** |
|  |  | 10 µM | **0.12** | **0.01** | **0.08** | **0.01** | **0.19** | **0.01** | **4.43** | **0.38** | **0.18** | **0.02** | **0.31** | **0.03** |
|  | **FLP-18-5** | 0.1 µM | **0.65** | **0.10** | 0.83 | 0.08 | **0.18** | **0.01** | **5.02** | **0.49** | **0.70** | **0.05** | **0.69*** | **0.09** |
|  |  | 10 µM | **0.37** | **0.06** | 0.97 | 0.08 | **0.18** | **0.03** | **6.28** | **0.52** | **0.22** | **0.03** | **0.68*** | **0.06** |
|  | **FLP-21** | 0.1 µM | **0.14** | **0.02** | **0.85** | **0.09** | **0.63** | **0.04** | **2.78** | **0.27** | **0.58** | **0.04** | **0.31** | **0.02** |
|  |  | 10 µM | **0.16** | **0.02** | **0.57** | **0.05** | **0.27** | **0.02** | **6.18** | **0.57** | **0.19** | **0.04** | **0.15** | **0.02** |
|  | **FLP-27** | 0.1 µM | **0.91** | **0.08** | 0.84 | 0.13 | **1.07** | **0.14** | 0.88 | 0.04 | 1.02 | 0.08 | **0.71** | **0.09** |
|  |  | 10 µM | **0.71** | **0.05** | 1.15 | 0.13 | **0.57** | **0.05** | 1.61 | 0.08 | 0.88 | 0.06 | **0.20** | **0.04** |
|  | **FLP-33** | 0.1 µM | 0.72 | 0.02 | 0.77 | 0.02 | 0.85 | 0.07 | 0.79 | 0.06 | 0.86 | 0.05 | **0.77** | **0.07** |
|  |  | 10 µM | 0.82 | 0.05 | 0.85 | 0.05 | 0.79 | 0.03 | 0.85 | 0.04 | 0.91 | 0.04 | **0.21** | **0.03** |
|  | **FLP-34-1** | 0.1 µM | **0.78** | **0.03** | 0.81 | 0.05 | 0.78 | 0.03 | **0.89** | **0.04** | 0.93 | 0.04 | **0.23** | **0.06** |
|  |  | 10 µM | **0.54** | **0.04** | 0.92 | 0.07 | 0.72 | 0.04 | **3.36** | **0.34** | 0.79 | 0.04 | **0.09** | **0.02** |
|  | **FLP-34-2** | 0.1 µM | 0.70 | 0.06 | 0.83 | 0.04 | 0.78 | 0.04 | 0.86 | 0.03 | **0.88** | **0.05** | **0.22** | **0.07** |
|  |  | 10 µM | 0.78 | 0.07 | 0.92 | 0.06 | 0.93 | 0.07 | 1.01 | 0.07 | **0.58** | **0.03** | **0.11** | **0.02** |
| **human** | **NPY** | 0.1 µM | 1.29 | 0.10 | 1.05 | 0.05 | 1.13 | 0.03 | 0.94 | 0.08 | **1.05** | **0.08** | **0.75*** | **0.08** |
|  |  | 10 µM | 1.30 | 0.14 | 1.32 | 0.04 | 1.32 | 0.05 | 1.33 | 0.11 | **0.64** | **0.08** | **0.71*** | **0.09** |
|  | **PP** | 0.1 µM | 1.21 | 0.15 | 1.02 | 0.03 | 1.05 | 0.03 | 0.86 | 0.07 | 1.01 | 0.08 | **0.37** | **0.06** |
|  |  | 10 µM | 1.49 | 0.18 | 1.25 | 0.05 | 1.14 | 0.04 | 0.96 | 0.08 | 1.00 | 0.09 | **0.14** | **0.04** |
|  | **PYY** | 0.1 µM | 1.12 | 0.12 | 0.95 | 0.07 | 0.99 | 0.05 | 0.94 | 0.09 | **0.89** | **0.10** | **0.66** | **0.09** |
|  |  | 10 µM | 1.19 | 0.07 | 1.20 | 0.05 | 1.14 | 0.05 | 1.01 | 0.03 | **0.21** | **0.03** | **0.23** | **0.04** |
|  | **PrRP20** | 0.1 µM | 1.14 | 0.15 | 1.00 | 0.04 | **1.10** | **0.07** | 0.90 | 0.11 | 0.96 | 0.10 | **0.90** | **0.10** |
|  |  | 10 µM | 1.45 | 0.18 | 1.23 | 0.07 | **0.67** | **0.06** | 1.05 | 0.14 | 1.19 | 0.14 | **0.65** | **0.09** |
|  | **PrRP31** | 0.1 µM | 1.30 | 0.19 | 1.10 | 0.03 | **1.05** | **0.05** | 0.91 | 0.05 | 0.96 | 0.06 | **1.06** | **0.19** |
|  |  | 10 µM | 1.41 | 0.17 | 1.29 | 0.04 | **0.64** | **0.03** | 1.10 | 0.11 | 1.25 | 0.14 | **0.74** | **0.11** |
|  | **NPFF** | 0.1 µM | 1.27 | 0.19 | 0.98 | 0.05 | **1.00** | **0.07** | 1.04 | 0.11 | **1.08** | **0.14** | 0.95 | 0.14 |
|  |  | 10 µM | 0.80 | 0.09 | 1.00 | 0.05 | **0.44** | **0.03** | 1.65 | 0.14 | **0.43** | **0.03** | 0.78 | 0.06 |
|  | **QRFP26** | 0.1 µM | 1.17 | 0.15 | 0.90 | 0.04 | **0.89** | **0.03** | **0.84** | **0.07** | 0.97 | 0.08 | **0.14** | **0.05** |
|  |  | 10 µM | 0.84 | 0.10 | 0.97 | 0.04 | **0.34** | **0.02** | **2.19** | **0.25** | 0.93 | 0.09 | **0.06** | **0.02** |

**Table S7. Cross-species reactivity - cAMP assay data of human receptors.** Legend of Table S6 applies.

|  |  |  | **Y_1_R** | | **Y_2_R** | | **Y_4_R** | | **Y_5_R** | | **NPFF_1_R** | | **NPFF_2_R** | |
| --- | --- | --- | --- | --- | --- | --- | --- | --- | --- | --- | --- | --- | --- | --- |
|  |  |  | mean | ±SEM | mean | ±SEM | mean | ±SEM | mean | ±SEM | mean | ±SEM | mean | ±SEM |
| ***C. elegans*** | **FLP-21** | 0.1 µM | 0.91 | 0.06 | **0.78** | **0.09** | **0.91** | **0.02** | **0.84** | **0.05** | **0.38** | **0.04** | **0.17** | **0.02** |
|  |  | 10 µM | 0.80 | 0.03 | **0.13** | **0.04** | **0.34** | **0.04** | **0.25** | **0.04** | **0.43** | **0.02** | **0.47** | **0.02** |
|  | **FLP-27** | 0.1 µM | **0.88** | **0.12** | **0.80** | **0.08** | **1.01** | **0.15** | 1.07 | 0.16 | **0.76** | **0.08** | **0.66** | **0.05** |
|  |  | 10 µM | **0.23** | **0.03** | **0.17** | **0.03** | **0.75** | **0.11** | 0.96 | 0.11 | **0.26** | **0.04** | **0.18** | **0.03** |
|  | **FLP-33** | 0.1 µM | 0.94 | 0.08 | 0.89 | 0.08 | 0.95 | 0.05 | 0.94 | 0.10 | **0.81** | **0.04** | **0.82** | **0.04** |
|  |  | 10 µM | 0.73 | 0.06 | 0.76 | 0.06 | 0.90 | 0.05 | 0.99 | 0.09 | **0.47** | **0.01** | **0.16** | **0.02** |
|  | **FLP-34-1** | 0.1 µM | **0.99** | **0.07** | **0.79** | **0.05** | **0.67** | **0.02** | 0.98 | 0.08 | **0.89** | **0.03** | **0.86** | **0.05** |
|  |  | 10 µM | **0.70** | **0.04** | **0.23** | **0.05** | **0.08** | **0.01** | 0.77 | 0.04 | **0.73** | **0.02** | **0.35** | **0.04** |
|  | **FLP-34-2** | 0.1 µM | 0.94 | 0.09 | **0.85** | **0.07** | **0.88** | **0.05** | 0.96 | 0.10 | **0.79** | **0.05** | **0.69** | **0.04** |
|  |  | 10 µM | 1.01 | 0.12 | **0.42** | **0.05** | **0.33** | **0.03** | 1.14 | 0.10 | **0.21** | **0.02** | **0.13** | **0.01** |
| **human** | **NPY** | 0.1 µM | **0.03** | **0.01** | **0.05** | **0.02** | **0.14** | **0.03** | **0.07** | **0.02** | **0.78** | **0.07** | **0.86** | **0.09** |
|  |  | 10 µM | **0.02** | **0.01** | **0.26** | **0.05** | **0.07** | **0.03** | **0.11** | **0.04** | **0.74** | **0.09** | **0.52** | **0.11** |

**Table S8. Cross-species reactivity – IP accumulation assay data of *C. elegans* receptors.** Receptor activation was confirmed by inositol phosphate accumulation in transiently transfected HEK293 cells using the chimeric Gα_Δ6qi4myr_ to redirect signaling to the inositol phosphate pathway. Receptor activation by a peptide (IP > 2-fold and statistically significant from basal values as tested by one way ANOVA with Dunnett's post-hoc test and p values ≤ 0.05) is highlighted in bold. Negative controls were transfected with empty vector (mock) instead of the receptor and displayed no significant activation by any of the tested peptides. Receptors are arranged horizontally, peptides vertically. Data are shown as x-fold of basal (mean ± SEM) of at least three independent experiments.

|  |  | **NPR-1** | | **NPR-3** | | **NPR-4b** | | **NPR-5b** | | **NPR-6** | | **NPR-11** | | **mock** | |
| --- | --- | --- | --- | --- | --- | --- | --- | --- | --- | --- | --- | --- | --- | --- | --- |
|  |  | mean | ±SEM | mean | ±SEM | mean | ±SEM | mean | ±SEM | mean | ±SEM | mean | ±SEM | mean | ±SEM |
| **w/o** | / | 1.00 | 0.09 | 1.00 | 0.13 | 1.00 | 0.13 | 1.00 | 0.16 | 1.00 | 0.12 | 1.00 | 0.11 | 1.00 | 0.23 |
| **FLP-1** | 0.1 µM | 1.03 | 0.10 | 0.96 | 0.11 | **2.48** | **0.24** | 0.99 | 0.11 | **1.14** | **0.16** | **0.92** | **0.20** | 4.14 | 2.68 |
|  | 10 µM | 0.98 | 0.10 | 0.93 | 0.08 | **8.55** | **0.83** | 1.80 | 0.28 | **5.19** | **0.43** | **6.48** | **0.63** | 1.78 | 1.07 |
| **FLP-3-4** | 0.1 µM | 0.98 | 0.15 | 0.97 | 0.08 | **4.77** | **0.55** | **1.19** | **0.16** | **1.04** | **0.14** | 0.92 | 0.07 | 1.08 | 0.29 |
|  | 10 µM | 0.94 | 0.11 | 0.91 | 0.07 | **11.46** | **1.26** | **8.18** | **0.76** | **5.43** | **0.54** | 1.17 | 0.05 | 1.16 | 0.46 |
| **FLP-4-2** | 0.1 µM | 0.83 | 0.08 | 0.92 | 0.09 | **4.71** | **0.49** | 0.90 | 0.12 | 0.96 | 0.12 | **1.03** | **0.05** | 0.75 | 0.17 |
|  | 10 µM | 0.89 | 0.08 | 1.05 | 0.13 | **12.66** | **0.93** | 1.98 | 0.27 | 1.19 | 0.14 | **2.79** | **0.27** | 0.75 | 0.18 |
| **FLP-5-1** | 0.1 µM | 0.88 | 0.08 | 0.88 | 0.07 | **2.00** | **0.81** | 0.91 | 0.12 | 0.96 | 0.12 | **1.03** | **0.05** | 1.08 | 0.44 |
|  | 10 µM | 0.93 | 0.10 | 0.95 | 0.08 | **5.53** | **0.68** | 1.45 | 0.19 | 0.98 | 0.13 | **6.49** | **0.43** | 1.01 | 0.29 |
| **FLP-14** | 0.1 µM | 0.90 | 0.07 | 0.93 | 0.09 | **1.62** | **0.23** | 0.87 | 0.11 | **1.05** | **0.13** | **1.55** | **0.24** | 1.02 | 0.27 |
|  | 10 µM | 0.97 | 0.10 | 0.98 | 0.11 | **7.01** | **0.65** | 1.05 | 0.15 | **4.86** | **0.42** | **11.94** | **2.19** | 1.62 | 0.60 |
| **FLP-15-2** | 0.1 µM | 0.94 | 0.08 | **1.40** | **0.16** | **4.58** | **0.40** | **1.04** | **0.13** | **1.02** | **0.15** | **1.19** | **0.08** | 0.79 | 0.23 |
|  | 10 µM | 1.01 | 0.07 | **2.31** | **0.26** | **12.19** | **0.89** | **7.52** | **0.64** | **7.37** | **0.57** | **12.88** | **1.21** | 0.90 | 0.22 |
| **FLP-18-5** | 0.1 µM | 0.87 | 0.07 | 0.91 | 0.12 | **11.51** | **0.97** | **8.80** | **0.86** | **1.26** | **0.16** | **0.81** | **0.04** | 2.62 | 1.67 |
|  | 10 µM | 0.87 | 0.09 | 1.00 | 0.05 | **14.41** | **1.01** | **12.08** | **0.99** | **8.17** | **0.59** | **3.73** | **0.34** | 1.23 | 0.42 |
| **FLP-21** | 0.1 µM | 0.99 | 0.14 | 0.95 | 0.07 | **3.22** | **0.34** | **4.71** | **0.68** | **1.47** | **0.20** | **7.35** | **0.54** | 1.64 | 0.51 |
|  | 10 µM | 0.90 | 0.07 | 0.96 | 0.08 | **10.86** | **0.93** | **12.20** | **0.90** | **9.35** | **1.00** | **20.41** | **1.75** | 1.16 | 0.47 |

**Table S9. Cross-species reactivity – IP accumulation assay data of human receptors.** Receptor activation of selected peptides was determined by inositol phosphate accumulation in transiently transfected HEK293 cells. PrRPR and QRFPR endogenously couple to members of the Gα_q_ family (indicated by #), while for the other receptors a chimeric Gα_Δ6qi4myr_ was co-transfected to redirect signaling to the inositol phosphate pathway. Data are shown as x-fold of basal (mean ± SEM) of at least three independent experiments. Receptor activation by a peptide (IP > 2-fold and statistically significant from buffer control as tested by one way ANOVA with Dunnett's post-hoc test and p values ≤ 0.05) is highlighted in bold.

|  |  |  | **Y_1_R** | | **Y_2_R** | | **Y_4_R** | | **Y_5_R** | | **PrRPR#** | | **QRFPR#** | | **NPFF_1_R** | | **NPFF_2_R** | |
| --- | --- | --- | --- | --- | --- | --- | --- | --- | --- | --- | --- | --- | --- | --- | --- | --- | --- | --- |
|  |  |  | mean | ±SEM | mean | ±SEM | mean | ±SEM | mean | ±SEM | mean | ±SEM | mean | ±SEM | mean | ±SEM | mean | ±SEM |
|  | **w/o** | / | 1.00 | 0.15 | 1.00 | 0.10 | 1.00 | 0.13 | 1.00 | 0.27 | 1.00 | 0.12 | 1.00 | 0.27 | 1.00 | 0.10 | 1.00 | 0.13 |
| ***C. elegans*** | **FLP-1** | 0.1 µM | 1.25 | 0.33 | 2.81 | 1.41 | 1.28 | 0.33 | 1.33 | 0.79 | 1.04 | 0.27 | 1.07 | 0.16 | **2.11** | **0.36** | **1.10** | **0.19** |
|  |  | 10 µM | 3.68 | 2.37 | 1.41 | 0.35 | 1.45 | 0.29 | 1.17 | 0.25 | 1.27 | 0.24 | 0.92 | 0.20 | **3.54** | **0.53** | **2.86** | **0.24** |
|  | **FLP-3-4** | 0.1 µM | 1.54 | 0.63 | 1.01 | 0.25 | 1.31 | 0.23 | 0.67 | 0.22 | 1.86 | 0.81 | 1.42 | 0.36 | **1.29** | **0.11** | **1.58** | **0.27** |
|  |  | 10 µM | 3.82 | 1.09 | 0.70 | 0.12 | 1.29 | 0.33 | 1.41 | 0.36 | 0.96 | 0.14 | 1.52 | 0.43 | **3.42** | **0.59** | **3.28** | **0.25** |
|  | **FLP-4-2** | 0.1 µM | 2.37 | 0.78 | **0.76** | **0.08** | 1.45 | 0.28 | 1.14 | 0.30 | 0.85 | 0.13 | 1.26 | 0.28 | **1.86** | **0.52** | **1.07** | **0.24** |
|  |  | 10 µM | 1.93 | 0.44 | **2.05** | **0.29** | 1.04 | 0.16 | 1.29 | 0.20 | 0.86 | 0.16 | 0.98 | 0.19 | **3.68** | **0.81** | **2.36** | **0.51** |
|  | **FLP-5-1** | 0.1 µM | 1.02 | 0.29 | **0.88** | **0.17** | 1.38 | 0.32 | **0.80** | **0.19** | 1.20 | 0.30 | 0.95 | 0.18 | **1.48** | **0.38** | **0.88** | **0.25** |
|  |  | 10 µM | 1.69 | 0.76 | **4.08** | **0.73** | 1.28 | 0.33 | **5.88** | **0.93** | 1.41 | 0.55 | 1.21 | 0.30 | **4.07** | **0.67** | **2.44** | **0.43** |
|  | **FLP-14** | 0.1 µM | 0.71 | 0.33 | **1.05** | **0.07** | **0.97** | **0.19** | **0.96** | **0.28** | 1.07 | 0.08 | 1.01 | 0.13 | **3.87** | **1.08** | **2.10** | **0.34** |
|  |  | 10 µM | 2.18 | 0.42 | **4.60** | **1.07** | **3.01** | **0.33** | **13.09** | **2.54** | 0.70 | 0.17 | 1.16 | 0.34 | **4.07** | **0.30** | **4.12** | **0.88** |
|  | **FLP-15-2** | 0.1 µM | 0.91 | 0.41 | **0.89** | **0.13** | 1.46 | 0.48 | **1.10** | **0.35** | 0.93 | 0.21 | 0.83 | 0.10 | **2.72** | **0.24** | **3.03** | **0.39** |
|  |  | 10 µM | 1.41 | 0.35 | **5.21** | **0.37** | 1.73 | 0.19 | **2.71** | **0.62** | 1.13 | 0.38 | 1.42 | 0.29 | **4.54** | **1.26** | **3.72** | **0.82** |
|  | **FLP-18-5** | 0.1 µM | 4.51 | 2.96 | **1.43** | **0.59** | 1.86 | 0.62 | 0.69 | 0.14 | 1.27 | 0.34 | 1.76 | 0.82 | **2.31** | **0.26** | **1.76** | **0.12** |
|  |  | 10 µM | 2.67 | 1.30 | **3.87** | **0.76** | 1.37 | 0.29 | 1.61 | 0.42 | 2.34 | 0.67 | 1.49 | 0.32 | **2.53** | **0.33** | **3.11** | **0.21** |
|  | **FLP-21** | 0.1 µM | 2.44 | 0.71 | **1.32** | **0.26** | **1.88** | **0.51** | **1.13** | **0.35** | 1.37 | 0.36 | **0.92** | **0.24** | **3.03** | **0.43** | **3.39** | **0.23** |
|  |  | 10 µM | 2.92 | 0.41 | **14.46** | **1.34** | **4.45** | **0.76** | **13.22** | **1.66** | 1.12 | 0.30 | **3.10** | **0.51** | **3.39** | **0.56** | **3.01** | **0.46** |
|  | **FLP-27** | 0.1 µM |  |  |  |  |  |  |  |  | 0.93 | 0.10 | 0.87 | 0.07 |  |  |  |  |
|  |  | 10 µM |  |  |  |  |  |  |  |  | 0.91 | 0.06 | 0.95 | 0.08 |  |  |  |  |
|  | **FLP-33** | 0.1 µM |  |  |  |  |  |  |  |  | 0.91 | 0.08 | 0.91 | 0.07 |  |  |  |  |
|  |  | 10 µM |  |  |  |  |  |  |  |  | 0.88 | 0.05 | 0.80 | 0.10 |  |  |  |  |
|  | **FLP-34-1** | 0.1 µM |  |  |  |  |  |  |  |  | 0.95 | 0.07 | 0.89 | 0.07 |  |  |  |  |
|  |  | 10 µM |  |  |  |  |  |  |  |  | 0.78 | 0.10 | 0.83 | 0.07 |  |  |  |  |
|  | **FLP-34-2** | 0.1 µM |  |  |  |  |  |  |  |  | 0.91 | 0.07 | 0.77 | 0.05 |  |  |  |  |
|  |  | 10 µM |  |  |  |  |  |  |  |  | 0.77 | 0.06 | 0.84 | 0.11 |  |  |  |  |
| **human** | **NPY** | 0.1 µM | **24.89** | **3.30** | **12.23** | **1.11** | **3.01** | **0.25** | **17.66** | **1.71** | 0.95 | 0.15 | 0.77 | 0.17 | 0.81 | 0.07 | **1.01** | **0.13** |
|  |  | 10 µM | **25.57** | **3.42** | **10.85** | **1.18** | **4.63** | **0.41** | **17.85** | **1.81** | 0.88 | 0.20 | 0.79 | 0.18 | 0.93 | 0.07 | **2.17** | **0.15** |
|  | **PP** | 0.1 µM | **17.01** | **2.19** | **3.40** | **0.11** | **4.73** | **0.44** | **16.39** | **1.77** | 0.99 | 0.13 | 0.75 | 0.14 | 0.75 | 0.08 | 0.95 | 0.14 |
|  |  | 10 µM | **25.89** | **3.08** | **12.24** | **1.17** | **4.61** | **0.45** | **17.90** | **1.92** | 1.01 | 0.15 | 0.75 | 0.10 | 0.75 | 0.10 | 1.44 | 0.15 |
|  | **PYY** | 0.1 µM | **25.80** | **2.79** | **11.48** | **1.13** | **3.90** | **0.47** | **17.55** | **1.92** | 0.95 | 0.11 | 0.70 | 0.10 | 0.75 | 0.08 | 0.93 | 0.13 |
|  |  | 10 µM | **24.73** | **2.82** | **10.17** | **1.16** | **4.46** | **0.45** | **17.60** | **1.84** | 0.88 | 0.13 | 0.65 | 0.06 | 0.81 | 0.08 | 1.91 | 0.15 |
|  | **PrRP20** | 0.1 µM | 0.82 | 0.10 | **0.81** | **0.12** | 0.80 | 0.14 | **1.01** | **0.38** | **26.01** | **2.52** | 0.59 | 0.08 | **0.94** | **0.06** | **1.75** | **0.11** |
|  |  | 10 µM | 2.68 | 0.97 | **5.05** | **1.41** | 0.89 | 0.16 | **5.90** | **0.62** | **27.38** | **2.59** | 0.63 | 0.10 | **2.37** | **0.07** | **2.32** | **0.18** |
|  | **PrRP31** | 0.1 µM | 0.87 | 0.13 | **0.76** | **0.09** | 0.84 | 0.12 | **0.70** | **0.10** | **25.49** | **2.40** | 0.58 | 0.09 | **0.87** | **0.09** | **1.83** | **0.17** |
|  |  | 10 µM | 1.86 | 0.20 | **5.57** | **0.39** | 1.10 | 0.20 | **9.64** | **1.31** | **25.47** | **2.13** | 0.59 | 0.08 | **2.26** | **0.14** | **2.28** | **0.17** |
|  | **NPFF** | 0.1 µM | 0.90 | 0.14 | 0.76 | 0.13 | 0.89 | 0.14 | 0.59 | 0.10 | 0.89 | 0.19 | 0.58 | 0.09 | **1.76** | **0.16** | **2.33** | **0.16** |
|  |  | 10 µM | 0.99 | 0.15 | 0.98 | 0.15 | 0.99 | 0.14 | 1.11 | 0.19 | 1.28 | 0.10 | 0.67 | 0.11 | **2.44** | **0.11** | **2.28** | **0.16** |
|  | **QRFP26** | 0.1 µM | 1.32 | 0.22 | **1.30** | **0.20** | 1.03 | 0.15 | **0.87** | **0.14** | 1.06 | 0.15 | **22.77** | **1.10** | **1.04** | **0.12** | **1.85** | **0.17** |
|  |  | 10 µM | 2.75 | 0.10 | **10.62** | **1.04** | 1.42 | 0.17 | **3.57** | **0.53** | 1.91 | 0.30 | **24.26** | **0.87** | **2.56** | **0.13** | **2.58** | **0.16** |

**Table S10. Influence of the conserved arginine (RxA mutation) of *C. elegans* FLP-15-2, FLP-21 and human PYY on receptor activity.** G_i/o_ activity was measured by a cAMP reporter gene assay in transiently transfected HEK293 cells. The inhibition of cAMP production induced by 1 µM forskolin (set to 1.00) was observed. For NPR-5b, an increase of cellular cAMP is detected, consistent with G_s_ coupling. Receptor activation of human peptides was determined by inositol phosphate accumulation using the chimeric Gα_Δ6qi4myr_ to redirect signaling to the inositol phosphate pathway in transiently transfected HEK293 cells. Data represent x-fold of basal (mean ± SEM) of at least three independent experiments.

|  | | | | ***C. elegans* receptors** | | | | | | | | | | | |
| --- | --- | --- | --- | --- | --- | --- | --- | --- | --- | --- | --- | --- | --- | --- | --- |
|  |  |  |  | **NPR-1** | | **NPR-3** | | **NPR-4b** | | **NPR-5b** | | **NPR-6** | | **NPR-11** | |
|  |  |  |  | mean | ±SEM | mean | ±SEM | mean | ±SEM | mean | ±SEM | mean | ±SEM | mean | ±SEM |
| ***C. elegans* peptides** | **G_i_ (G_s_) [x-fold of forskolin]** | **w/o** | / | 0.10 | 0.01 | 0.07 | 0.02 | 0.08 | 0.01 | 0.04 | 0.00 | 0.10 | 0.01 | 0.07 | 0.00 |
|  |  | **Forskolin** | 10 µM | 1.00 | 0.10 | 1.00 | 0.17 | 1.00 | 0.07 | 1.00 | 0.05 | 1.00 | 0.03 | 1.00 | 0.07 |
|  |  | **FLP-15-2** | 0.1 µM | 0.43 | 0.02 | 0.97 | 0.23 | 0.58 | 0.05 | 1.05 | 0.07 | 0.86 | 0.05 | 0.91 | 0.08 |
|  |  |  | 10 µM | 0.27 | 0.02 | 0.41 | 0.06 | 0.26 | 0.03 | 4.95 | 0.31 | 0.46 | 0.10 | 0.30 | 0.03 |
|  |  | **[R8A]FLP-15-2** | 0.1 µM | 0.84 | 0.09 | 0.97 | 0.18 | 0.93 | 0.07 | 0.98 | 0.06 | 0.92 | 0.08 | 0.93 | 0.06 |
|  |  |  | 10 µM | 0.85 | 0.10 | 0.88 | 0.14 | 0.97 | 0.10 | 0.93 | 0.06 | 0.99 | 0.08 | 0.53 | 0.04 |
|  |  | **FLP-21** | 0.1 µM | 0.28 | 0.04 | 1.03 | 0.23 | 0.76 | 0.07 | 2.52 | 0.20 | 0.71 | 0.07 | 0.42 | 0.05 |
|  |  |  | 10 µM | 0.30 | 0.05 | 1.24 | 0.23 | 0.40 | 0.04 | 7.63 | 0.32 | 0.43 | 0.10 | 0.14 | 0.01 |
|  |  | **[R8A]FLP-21** | 0.1 µM | 0.90 | 0.06 | 1.20 | 0.26 | 0.95 | 0.05 | 1.06 | 0.07 | 1.00 | 0.09 | 0.87 | 0.11 |
|  |  |  | 10 µM | 0.89 | 0.06 | 1.38 | 0.34 | 0.99 | 0.06 | 0.97 | 0.06 | 0.86 | 0.04 | 0.67 | 0.07 |
|  |  |  | | **human receptors** | | | | | | | | | | | |
|  |  |  |  | **Y_1_R** | | **Y_2_R** | | **Y_4_R** | | **Y_5_R** | | **NPFF_1_R** | | **NPFF_2_R** | |
|  |  | **w/o** | / | 0.10 | 0.01 | 0.14 | 0.02 | 0.10 | 0.02 | 0.10 | 0.01 | 0.06 | 0.00 | 0.07 | 0.01 |
|  |  | **Forskolin** | 10 µM | 1.00 | 0.10 | 1.00 | 0.06 | 1.00 | 0.11 | 1.00 | 0.04 | 1.00 | 0.07 | 1.00 | 0.08 |
|  |  | **FLP-15-2** | 0.1 µM | 0.48 | 0.10 | 0.63 | 0.14 | 0.95 | 0.07 | 0.85 | 0.17 | 0.56 | 0.11 | 0.22 | 0.02 |
|  |  |  | 10 µM | 0.76 | 0.09 | 0.34 | 0.05 | 0.86 | 0.14 | 0.72 | 0.06 | 0.53 | 0.08 | 0.67 | 0.10 |
|  |  | **[R8A]FLP-15-2** | 0.1 µM | 0.72 | 0.08 | 0.86 | 0.07 | 0.79 | 0.06 | 0.89 | 0.11 | 0.91 | 0.04 | 0.94 | 0.08 |
|  |  |  | 10 µM | 0.71 | 0.07 | 1.05 | 0.15 | 0.94 | 0.07 | 0.91 | 0.07 | 0.71 | 0.05 | 0.97 | 0.08 |
|  |  | **FLP-21** | 0.1 µM | 0.72 | 0.14 | 0.58 | 0.05 | 0.85 | 0.07 | 0.94 | 0.12 | 0.30 | 0.03 | 0.17 | 0.01 |
|  |  |  | 10 µM | 0.58 | 0.05 | 0.21 | 0.02 | 0.36 | 0.04 | 0.29 | 0.02 | 0.52 | 0.10 | 0.80 | 0.11 |
|  |  | **[R8A]FLP-21** | 0.1 µM | 0.85 | 0.10 | 0.88 | 0.11 | 0.81 | 0.11 | 0.84 | 0.07 | 1.03 | 0.10 | 0.88 | 0.06 |
|  |  |  | 10 µM | 0.76 | 0.06 | 0.81 | 0.06 | 0.85 | 0.10 | 0.87 | 0.08 | 0.66 | 0.04 | 0.60 | 0.06 |
| **human peptides** | **Gα_Δ6qi4myr_ [x-fold of basal]** |  | | **human receptors** | | | | | | | | ***C. elegans* receptors** | | | |
|  |  |  |  | **Y_1_R** | | **Y_2_R** | | **Y_4_R** | | **Y_5_R** | | **NPR-6** | | **NPR-11** | |
|  |  | **w/o** | / | 1.00 | 0.06 | 1.00 | 0.07 | 1.00 | 0.18 | 1.00 | 0.10 | 1.00 | 0.05 | 1.00 | 0.10 |
|  |  | **PYY** | 0.1 µM | 24.18 | 1.75 | 14.65 | 1.02 | 4.14 | 0.75 | 20.02 | 1.97 | 0.93 | 0.06 | 1.39 | 0.24 |
|  |  |  | 10 µM | 21.22 | 3.24 | 11.36 | 1.63 | 5.75 | 1.22 | 17.17 | 2.62 | 3.80 | 0.29 | 7.68 | 2.55 |
|  |  | **[R35A]PYY** | 0.1 µM | 1.93 | 0.35 | 4.99 | 1.78 | 0.88 | 0.22 | 1.06 | 0.20 | 1.04 | 0.10 | 1.41 | 0.27 |
|  |  |  | 10 µM | 8.08 | 0.38 | 15.38 | 1.80 | 2.79 | 0.81 | 7.53 | 1.14 | 0.99 | 0.05 | 0.72 | 0.10 |

**Table S11. Potencies and efficacies of selected peptide-receptor pairs - cAMP assay data of *C. elegans* receptors.** For selected peptide-receptor pairs full concentration-response curves were determined via a cAMP reporter gene assay in transiently transfected HEK293 cells. G_i/o_ activity was measured by the inhibition of cAMP production induced by additional 1 µM forskolin (set to 1.00) to the given peptide, i.e. lower values correspond to high G_i/o_ activity. Potencies and efficacies of [RxA] mutated peptides could not be determined since they show no receptor activation even with high concentrations and are thus not included in this table. For the corresponding concentration-response curves, see Figure S1. Data represent mean ± SEM of at least three independent experiments

| receptor | peptide | EC_50_ [nM]  (pEC_50_ ± SEM) | Emax ± SEM [%] |
| --- | --- | --- | --- |
| NPR-1 | FLP-14 | 24  (7.6 ± 0.57) | -0.51 ± 0.13 |
|  | FLP-15-2 | 22  (7.6 ± 0.24) | -0.92 ± 0.1 |
|  | FLP-18-5 | 1823  (5.4 ± 0.58) | -0.53 ± 0.20 |
|  | FLP-21 | 0.97  (9.0 ± 0.25) | -0.97 ± 0.15 |
|  | FLP-34-1 | 578  (6.2 ± 0.39) | -0.65 ± 0.14 |
| NPR-3 | FLP-15-2 | 56  (7.3 ± 0.23) | -0.80 ± 0.09 |
|  | FLP-21 | 50  (5.3 ± 0.53) | -0.59 ± 0.26 |
| NPR-4b | FLP-14 | 2430  (5.6 ± 0.39) | -0.77 ± 0.20 |
|  | FLP-15-2 | 170  (6.8 ± 0.24) | -0.65 ± 0.07 |
|  | FLP-18-5 | 3  (8.5 ± 0.16) | -0.60 ± 0.05 |
|  | FLP-21 | 358  (6.4 ± 0.26) | -0.58 ± 0.07 |
| NPR-5b | FLP-15-2 | 6653  (5.2 ± 0.57) | 3.28 ± 1.7 |
|  | FLP-18-5 | 65  (7.2 ± 0.13) | 3.6 ± 0.22 |
|  | FLP-21 | 432  (6.4 ± 0.14) | 3.8 ± 0.26 |
|  | FLP-34-1 | 20120  (4.7 ± 0.80) | 6.7 ± 8.21 |
| NPR-6 | FLP-14 | 1177  (5.9 ± 0.40) | -0.82 ± 0.20 |
|  | FLP-15-2 | 678  (6.2 ± 0.37) | -0.72 ± 0.14 |
|  | FLP-18-5 | 425  (6.4 ± 0.38) | -0.70 ± 0.13 |
|  | FLP-21 | 409  (6.4 ± 0.26) | -0.65 ± 0.08 |
|  | NPY | 1358  (5.9 ± 0.25) | -0.43 ± 0.25 |
|  | PYY | 346  (6.5 ± 0.32) | -0.60 ± 0.09 |
| NPR-11 | FLP-14 | 1892  (5.7 ± 0.35) | -0.86 ± 0.19 |
|  | FLP-15-2 | 998  (6.0 ± 0.26) | -0.76 ± 0.11 |
|  | FLP-18-5 | 88  (7.1 ± 1.03) | -0.16 ± 0.08 |
|  | FLP-21 | 102  (7.0 ± 0.18) | -0.72 ± 0.06 |
|  | FLP-33 | 414  (6.6 ± 0.21) | -0.57 ± 0.06 |
|  | FLP-34-1 | 19  (7.7 ± 0.35) | -0.62 ± 0.10 |
|  | FLP-34-2 | 0.7  (9.2 ± 0.35) | -0.65 ± 0.14 |
|  | NPY | 96  (7.0 ± 0.49) | -0.32 ± 0.07 |
|  | PYY | 24  (7.6 ± 0.29) | -0.61 ± 0.08 |

**Figure S1. Concentration-response curves of selected peptide-receptor pairs - cAMP assay data of *C. elegans* receptors.** For selected peptide-receptor pairs concentration-response curves were determined via a cAMP reporter gene assay in transiently transfected HEK293 cells. G_i/o_ activity was measured by the inhibition of cAMP production induced by additional 1 µM forskolin (set to 1.00) to the given peptide, i.e. lower values correspond to high G_i/o_ activity. For the corresponding EC_50_ values (potency), see Table S9. Data represent x‑fold of forskolin (mean ± SEM) of at least three independent experiments

**
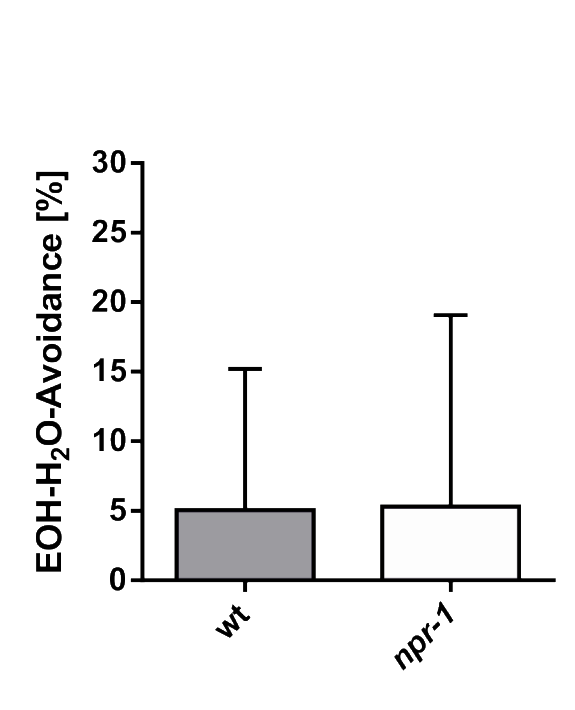
**

**Figure S2. Effect of ethanol on *C. elegans* MeSa aviodance.** Ethanol has no detectable effect on *C. elegans* compared to water in the chemotaxis assay employed in this study. Data are presented as mean ± SD in at least three independent experiments (n ≥ 1000).

**
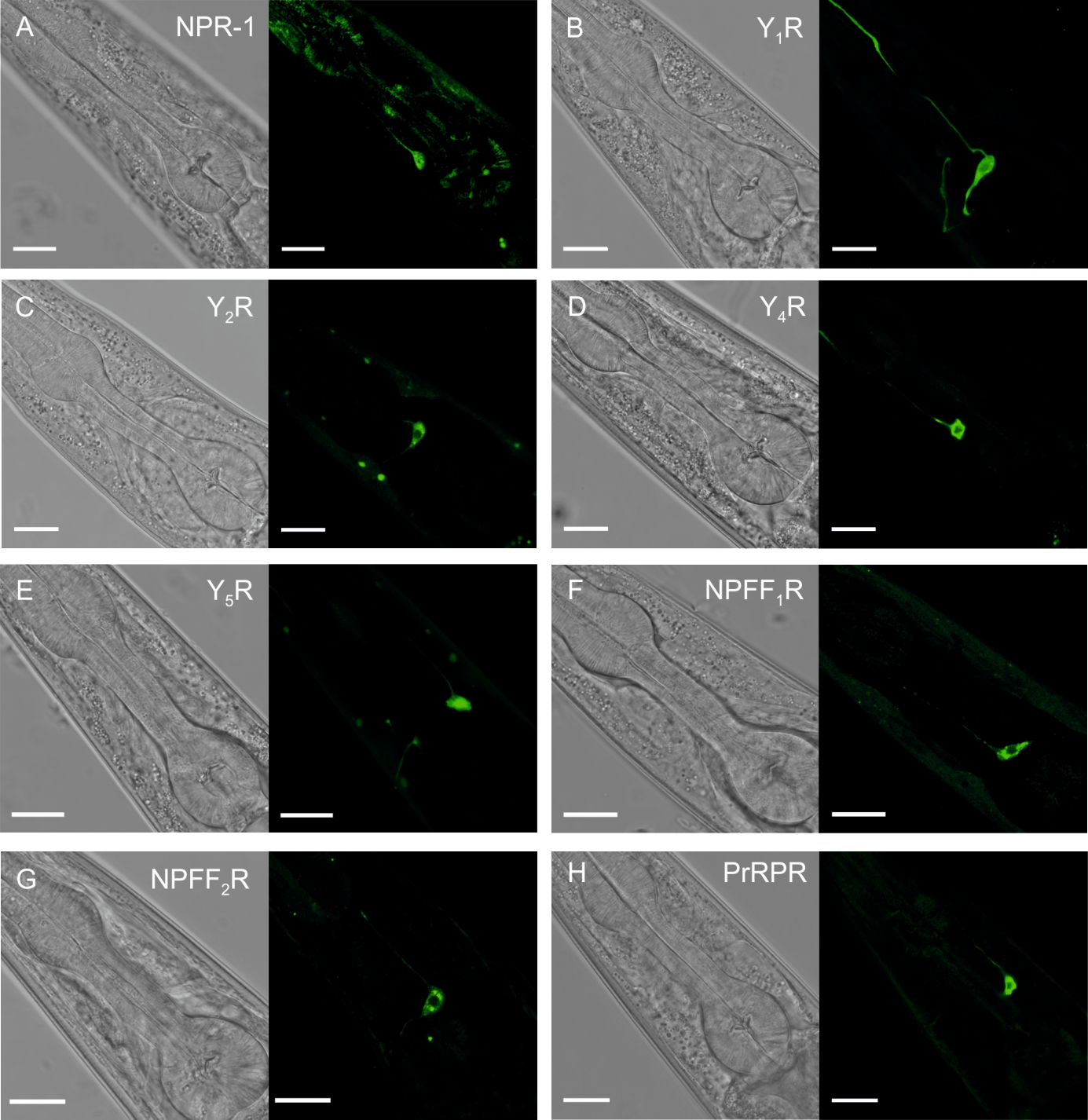
**

**Figure S3. Expression of human neuropeptide receptors in *C. elegans*.** Expression of endogenous NPR-1 (A) and human neuropeptide receptors (B-H) under the control of a 2 kb *npr-1* promoter fused to GFP in *C. elegans* head neurons. Although expression of npr-1 appears stronger, the localization pattern of the human GPCRs is indistinguishable. Displayed is the pharynx of adult worms, widefield (left) and fluorescence (right). Scale bars = 15 µm.

**
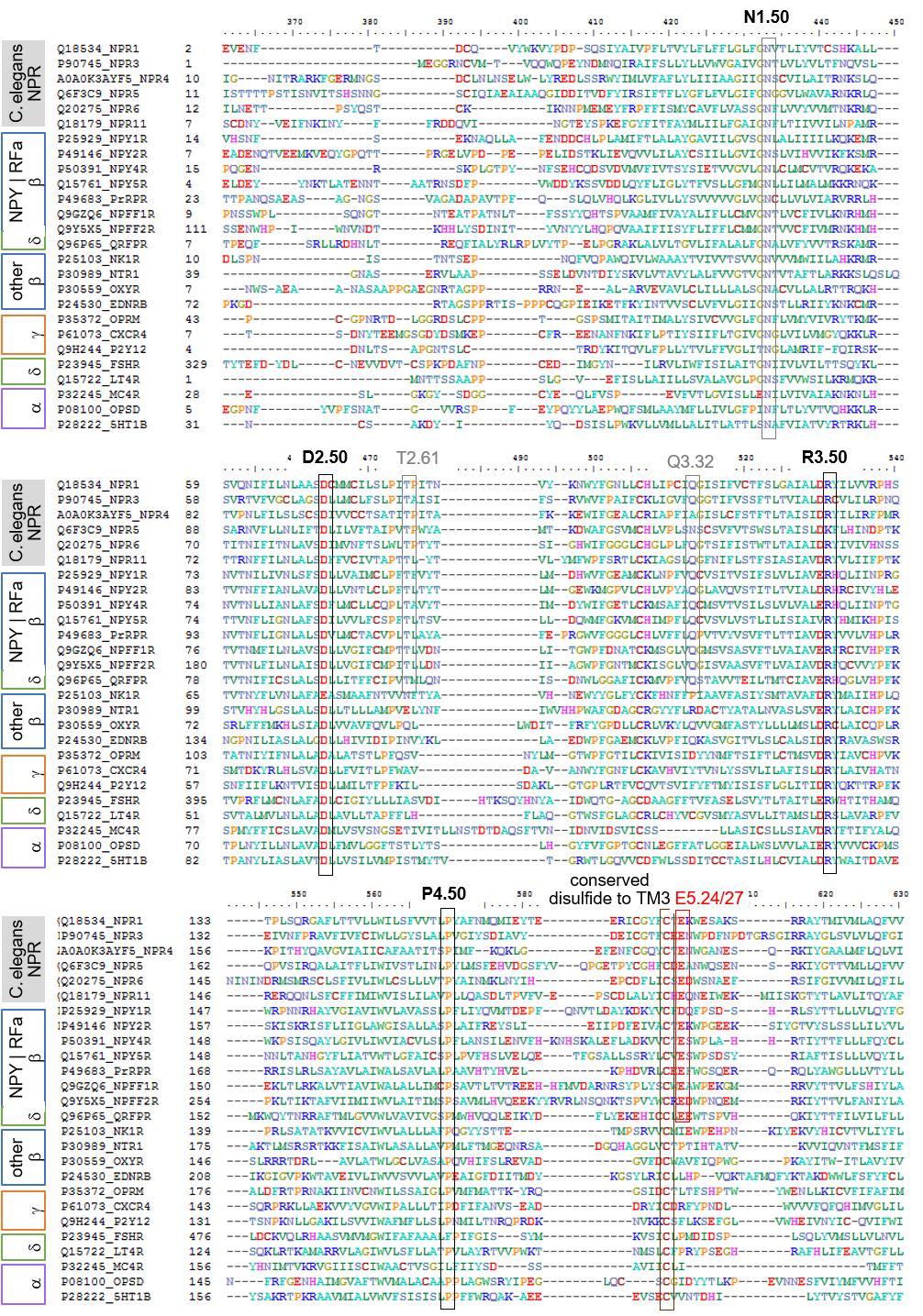
**

Figure S4 (continued on next page)

**
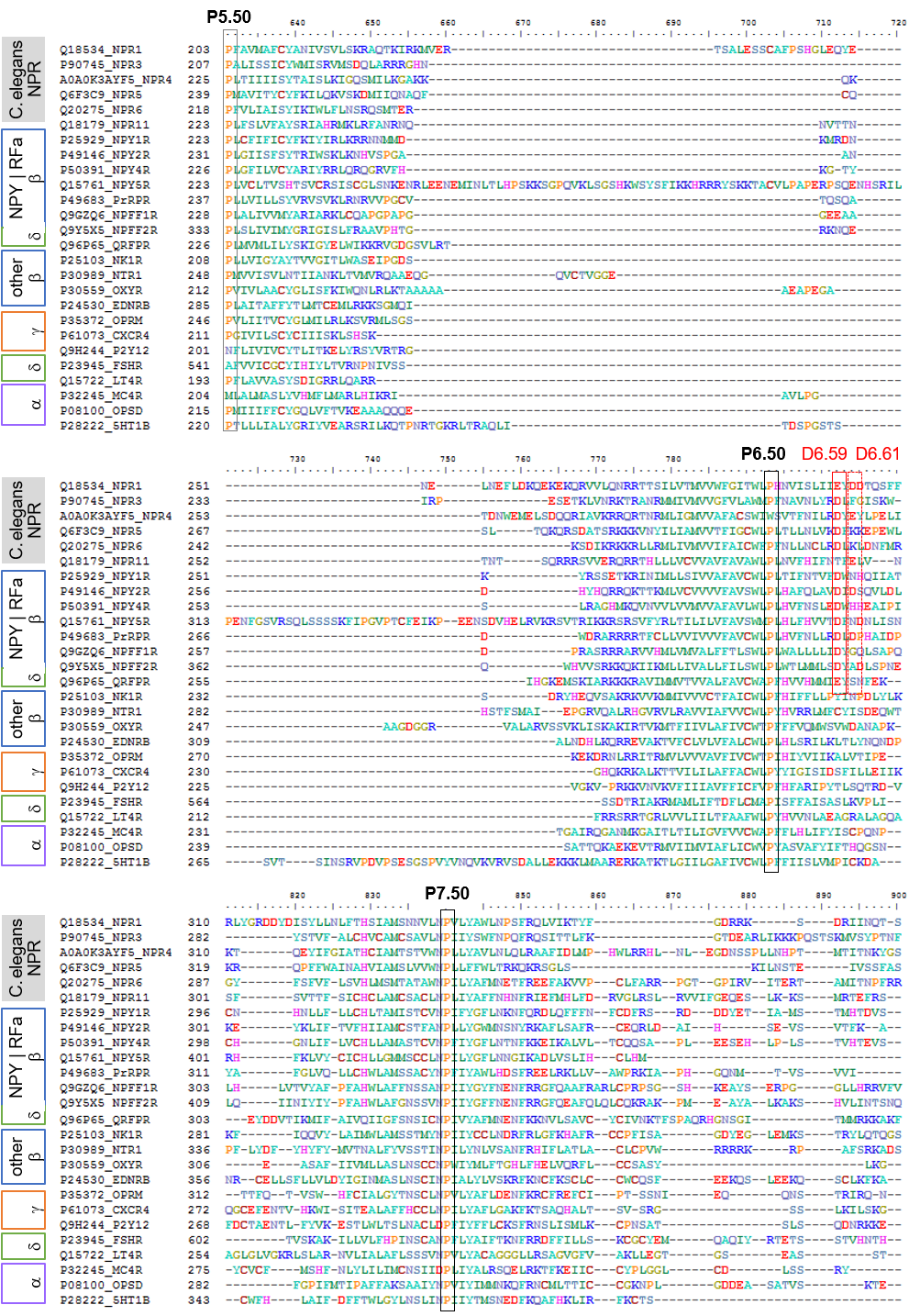
 Figure S4. Alignment of *C. elegans* NPRs with selected human GPCRs.** *C. elegans* NPRs are aligned against human NPY and other RFamide receptors which belong to the β-branch of GPCRs (according to [3]) except for QRFPR (δ), as well as other mainly peptide-binding GPCRs from the β, γ, δ or α-branch. Rhodopsin (OPSD), serotonin (5HT1B, both α) as well as leukotriene B4 (LT4R, γ) and purine (P2Y12, δ) receptors represent outgroups not activated by peptidic ligands. NPY and RFa receptors display conserved positions T2.61, Q3.32, E5.24/5.27 (ECL2) and D6.59 that are engaged in ligand binding to the human receptors [4-6], and are also found in *C. elegans* NPRs. NPR-11 is the only receptor that does not have an aspartate at position 6.59, but slightly displaced at position 6.61 which also shows an enrichment of aspartate among NPY/RFa receptors. Conserved transmembrane residues are marked according to the Ballesteros and Weinstein nomenclature [7] in bold and black rectangles. Codes represent Uniprot identifier. Distal N‑ and C-termini are omitted from the alignment for clarity. Alignments were generated with ClustalOmega [8].

**Supplementary References**

1. Brenner S: **The genetics of Caenorhabditis elegans**. *Genetics* 1974, **77**(1):71-94.

2. de Bono M, Bargmann CI: **Natural Variation in a Neuropeptide Y Receptor Homolog Modifies Social Behavior and Food Response in C. elegans**. *Cell* 1998, **94**(5):679-689.

3. Fredriksson R, Lagerstrom MC, Lundin LG, Schioth HB: **The G-protein-coupled receptors in the human genome form five main families. Phylogenetic analysis, paralogon groups, and fingerprints**. *Mol Pharmacol* 2003, **63**(6):1256-1272.

4. Merten N, Lindner D, Rabe N, Rompler H, Morl K, Schoneberg T, Beck-Sickinger AG: **Receptor subtype-specific docking of Asp6.59 with C-terminal arginine residues in Y receptor ligands**. *J Biol Chem* 2007, **282**(10):7543-7551.

5. Kaiser A, Muller P, Zellmann T, Scheidt HA, Thomas L, Bosse M, Meier R, Meiler J, Huster D, Beck-Sickinger AG *et al*: **Unwinding of the C-Terminal Residues of Neuropeptide Y is critical for Y(2) Receptor Binding and Activation**. *Angew Chem Int Ed Engl* 2015, **54**(25):7446-7449.

6. Yang Z, Han S, Keller M, Kaiser A, Bender BJ, Bosse M, Burkert K, Kogler LM, Wifling D, Bernhardt G *et al*: **Structural basis of ligand binding modes at the neuropeptide Y Y1 receptor**. *Nature* 2018, **556**(7702):520-524.

7. Ballesteros JA, Weinstein H: **Integrated methods for the construction of three-dimensional models and computational probing of structure-function relations in G protein-coupled receptors**. In: *Methods in Neurosciences.* Edited by Sealfon SC, vol. 25: Elsevier; 1995: 366-428.

8. Sievers F, Wilm A, Dineen D, Gibson TJ, Karplus K, Li W, Lopez R, McWilliam H, Remmert M, Soding J *et al*: **Fast, scalable generation of high-quality protein multiple sequence alignments using Clustal Omega**. *Mol Syst Biol* 2011, **7**:539.
